# Fatty acids derived from the probiotic *Lacticaseibacillus rhamnosus* HA-114 suppress age-dependent neurodegeneration

**DOI:** 10.1038/s42003-022-04295-8

**Published:** 2022-12-07

**Authors:** Audrey Labarre, Ericka Guitard, Gilles Tossing, Anik Forest, Eric Bareke, Marjorie Labrecque, Martine Tétreault, Matthieu Ruiz, J. Alex Parker

**Affiliations:** 1 Département de Neurosciences, Université de Montréal, Montréal, QC Canada; 2 Centre de Recherche du Centre Hospitalier de l’Université de Montréal (CRCHUM), Montréal, QC Canada; 3 Centre de Recherche, Institut de Cardiologie de Montréal, Montréal, QC Canada; 4 Département de Biochimie, Université de Montréal, Montréal, QC Canada; 5 Département de Nutrition, Université de Montréal, Montréal, QC Canada

**Keywords:** Amyotrophic lateral sclerosis, Amyotrophic lateral sclerosis, Behavioural genetics

## Abstract

The human microbiota is believed to influence health. Microbiome dysbiosis may be linked to neurological conditions like Alzheimer’s disease, amyotrophic lateral sclerosis, and Huntington’s disease. We report the ability of a probiotic bacterial strain in halting neurodegeneration phenotypes. We show that *Lacticaseibacillus rhamnosus* HA-114 is neuroprotective in *C. elegans* models of amyotrophic lateral sclerosis and Huntington’s disease. Our results show that neuroprotection from *L. rhamnosus* HA-114 is unique from other *L. rhamnosus* strains and resides in its fatty acid content. Neuroprotection by *L. rhamnosus* HA-114 requires *acdh-1/ACADS*B, *kat-1/ACAT1* and *elo-6/ELOVL3/6*, which are associated with fatty acid metabolism and mitochondrial β-oxidation. Our data suggest that disrupted lipid metabolism contributes to neurodegeneration and that dietary intervention with *L. rhamnosus* HA-114 restores lipid homeostasis and energy balance through mitochondrial β-oxidation. Our findings encourage the exploration of *L. rhamnosus* HA-114 derived interventions to modify the progression of neurodegenerative diseases.

## Abstract (web summary)

A probiotic bacterial strain (*Lacticaseibacillus rhamnosus*) delays neurodegeneration phenotypes in *C. elegans* models of amyotrophic lateral sclerosis and Huntington’s disease, with fatty acid extracts from the bacteria able to reproduce the protective effect.

## Introduction

The human body is the natural habitat for many microbes, including hundreds of bacterial species referred to as the microbiota. A growing body of evidence demonstrates that gut microbiota is essential to human health^1^, and a bacterial imbalance, termed dysbiosis, may be linked to many human diseases. It has been suggested that the oro-gastrointestinal tract microbiome may extend its effects beyond its niche and contribute systemically to various age-dependent diseases^2,3^. Indeed, a growing number of studies have identified perturbations in gut microbiome for several neurodegenerative disorders, including amyotrophic lateral sclerosis (ALS), multiple sclerosis (MS), Alzheimer’s (AD), and Parkinson’s disease (PD)^4–7^.

Therefore, positively regulating the microbiota–health relationship has attracted attention as preventative or therapeutic approach for many diseases, including neurodegenerative disorders^8,9^. Probiotic bacteria are defined as live microorganisms that may have positive health effects when consumed by the host. Emerging research has focused on using probiotic supplementation to counteract dysbiosis with the goal of stabilizing cognitive and emotional deficits in Alzheimer’s disease^10,11^. However, the characterization of similar effects in amyotrophic lateral sclerosis (ALS) models awaits investigation. Of interest are studies showing that antibiotic treatment depleting the microbiome, or treatment with specific molecules derived from bacteria, like γ-butyrate, can delay the onset of phenotypes in ALS in hSOD1^G93A^ and *C9orf72* loss-of-function mice^12–14^. However, insights into the molecular mechanisms underlying these effects are not fully understood.

Invertebrate model systems like *Caenorhabditis elegans* are maintained in the presence of bacteria in natural and laboratory settings. Aided by a wide range of genetic techniques it is possible to make direct connections between the worm’s bacterial diet, phenotypes and molecular mechanisms. Thus *C. elegans* has emerged as a suitable model to study effects of microbiota on life traits, gene expression, metabolic changes, and neuronal health^15–20^. Furthermore, it is also an established model for investigating conserved genetic pathways that regulate the cellular stress response and neurodegeneration^21–23^.

We used *C. elegans* to screen for the effects of dietary supplementation of a panel of probiotic bacteria combinations on neurodegenerative phenotypes. We identified *Lacticaseibacillus rhamnosus* HA-114 as a bacterial strain that suppressed motor phenotypes and neurodegeneration in simple genetic models of ALS. A combination of genetics, genome profiling, behavioral analysis and microscopy highlighted lipid homeostasis disruption as a potential mechanism driving neurodegeneration. Moreover, we found that the beneficial effect of *L. rhamnosus* HA-114 could be extended to other genetic models of age-dependent neurodegeneration, including Huntington’s disease (HD). We identified *acdh-1* and *kat-1*, genes implicated in fatty acid metabolism and β-oxidation, as the core components of this neuroprotective mechanism. Altogether, these results demonstrate that a dietary probiotic intervention potentially modulating microbiota composition may regulate the neurodegeneration process and provide benefits to the host by restoring energy balance.

## Results

### *L. rhamnosus* HA-114 harbors neuroprotective activity in age-dependent models of neurodegeneration

**Fig. 1:**
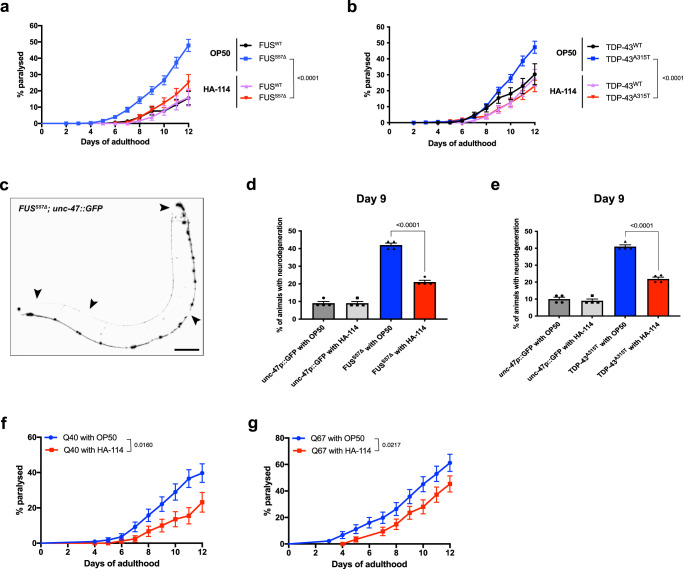
*Lacticaseibacillus rhamnosus* HA-114 rescues motor defects and neurodegeneration. Transgenics were monitored from the adult stage, scored daily for paralysis and fed with control OP50 or HA-114. **a** Mutant FUS worms fed with HA-114 showed less paralysis compared to transgenics expressing mutant FUS fed with OP50. **b** Transgenics expressing mutant TDP-43 fed with probiotics showed a lower rate of progressive paralysis than transgenics expressing mutant TDP-43 fed with OP50. **c** Image of a whole FUS^S57Δ^ worm expressing GFP in the GABAergic motor neurons. mFUS transgenics display gaps along neuronal processes (arrows). Scale bar = 100 μm. **d** Mutant FUS worms fed with HA-114 have a similar rate of neurodegeneration compared to transgenic GFP controls at day 9. **e** Mutant TDP-43 transgenics fed with probiotics had a lower rate of neurodegeneration at day 9 compared to mutant TDP-43 transgenics fed with OP50. **f** HA-114 rescued aged-dependent paralysis phenotype in transgenics expressing Q40 and in Q67 (G) animals. For paralysis assays (**a**, **b**, **f**, **g**), curves were generated and compared using the log-rank (Mantel–Cox) test. **a**: FUS^WT^ on OP50 *n* = 196; FUS^S57Δ^ on OP50 *n* = 407; FUS^WT^ on HA-114 *n* = 222; FUS^S57Δ^ on HA-114 *n* = 235. **b**: TDP-43^WT^ on OP50 *n* = 238; TDP-43^A315T^ on OP50 *n* = 526; TDP-43^WT^ on HA-114 *n* = 217; TDP-43^A315T^ on HA-114 *n* = 437. **f**: 40Q on OP50 *n* = 110; 40Q on HA-114 *n* = 90. **g**: 67Q on OP50 *n* = 90; 67Q on HA-114 *n* = 90. For neurodegeneration assays (**d**, **e**), one-way ANOVA were performed. For each conditions, *n* = 4 (25 worms per *n*). Data are presented as mean ± SEM.

We previously created *C. elegans* ALS strains expressing full-length, untagged, human FUS or TDP-43 protein mutants in motor neurons, under the *unc-47* promoter^24^. Transgenic worms with motor neuron-specific expression of these mutant proteins show age-dependent paralysis and GABAergic degeneration at a rate significantly higher than wild-type FUS or TDP-43 expressing strains^25^. These phenotypes, associated with the disease, typically develop over 6–12 days of adulthood for worms cultured on petri plates and fed with *E. coli* OP50, their regular food source in a laboratory setting. To investigate whether dietary probiotic interventions could modulate the phenotypes associated with ALS in our transgenic *C. elegans* models, we screened 16 different probiotic formulations, including 13 individual strains and 3 combinations (Table 1). We found that *L. rhamnosus* HA-114 rescued paralysis phenotypes on solid media in both of our ALS models, TDP-43^A315T^ and FUS^S57Δ^, while having no significant effect on TDP-43^WT^ and FUS^WT^ animals (Fig. 1a, b). Other probiotic strains and combinations had no or very little effect on paralysis phenotypes in FUS^S57Δ^ animals (Supplementary Fig. 1a–c). We also tested combinations of OP50 and HA-114 at different ratios (OP50:HA-114; 1:1, 3:1 and 1:3) and observed that all ratios slightly prevent paralysis (Supplementary Fig. 1d). Both 1:1 and 3:1 ratios slightly decreased paralysis phenotypes in FUS^S57Δ^ animal, while the 1:3 ratio showed the best results. However, none of the ratios tested had the same ability as HA-114 alone to prevent paralysis phenotype in our ALS worm models.

**Table 1:** List of probiotics from Lallemand Health Solutions.

Individual bacterial strains
*Bifidobacterium animalis subsp. lactis* B94
*Bifidobacterium breve* HA-129
*Bacillus subtilis* R0179
*Lacticaseibacillus plantarum* R1012
*Lacticaseibacillus plantarum* HA-119
*Lacticaseibacillus casei* L26
*Lacticaseibacillus paracasei* HA-196
*Lacticaseibacillus helveticus* R0052
*Lacticaseibacillus rhamnosus* R0011
*Lacticaseibacillus rhamnosus* R0343
*Lacticaseibacillus rhamnosus* HA-114
*Lacticaseibacillus rhamnosus* HA-111
*Pediococcus acidilactici* R1001

Blends
*L. plantarum* HA-119 and *B. animalis subsp. lactis* B94 (1:1)
*L. rhamnosus* HA-114 and *B. animalis subsp. lactis* B94 (1 :1)
*L. rhamnosus* HA-114 and *L. plantarum* HA-119 (1 :1)

These results suggested that concentrated HA-114 might play an important role in its neuroprotective effect. HA-114 was the only *L. rhamnosus* strain with the ability to prevent paralysis in FUS^S57Δ^ worms (Supplementary Fig. 2a). Rescue of paralysis was also observed when worms were fed from day 6 of adulthood, at the usual onset of symptoms (Supplementary Fig. 2b). However, HA-114 did not provide any lifespan extension in our FUS^S57Δ^ worms (Supplementary Fig. 2c).

Next, we assessed whether the rescue of paralysis phenotypes was associated with GABAergic motor neuron function and found a significant decrease in motor neuron degeneration in our transgenic animals compared to animals fed with control OP50 bacteria (Fig. 1c–e). Since oxidative stress seems to play an important role in many neurodegenerative disorders, we wondered if *L. rhamnosus* HA-114 would be able to prevent damage related to chronic exposure to oxidative stress. We exposed our mutant FUS worms to 250 μM paraquat over 12 days. Chronic paraquat exposure increased paralysis phenotypes in FUS^S57Δ^ animals fed with OP50, but not in animals fed with HA-114 (Supplementary Fig. 3a). We then assessed if HA-114 could also prevent acute oxidative stress. *hsp-6::GFP* worms^26^ were exposed to 5 mM paraquat over 24 h. *L. rhamnosus* HA-114 failed to decrease GFP signal when compared to OP50 fed worms (Supplementary Fig. 3b). Interestingly, both ascorbic acid and *N*-acetyl cysteine (NAC), two antioxidant compounds, failed to prevent paralysis phenotypes in FUS^S57Δ^ animals (Supplementary Fig. 3c). These results suggest that *L. rhamnosus* HA-114 can overcome damage linked with chronic oxidative stress, but likely not through antioxidant properties.

To determine whether the neuroprotective effect conferred by *L. rhamnosus* HA-114 was exclusive to TDP-43 or FUS pathogenesis or could be extended to other models of neurodegenerative diseases, we tested various models of age-associated neurodegeneration, including models of Huntington’s disease. We found that that *L. rhamnosus* HA-114 was also able to rescue paralysis phenotypes in worms expressing pan-neuronal polyglutamine repeats (Q40 and Q67, disease alleles)^27,28^ (Fig. 1f, g). These data suggest that paralysis and neurodegeneration phenotypes can be modulated through dietary probiotic intervention in nematodes and that *L. rhamnosus* HA-114 is effective in rescuing these phenotypes in several age-dependent models of neurodegeneration.

### Classic metabolic and stress pathways in *C. elegans* are not implicated in HA-114-mediated neuroprotection

**Fig. 2:**
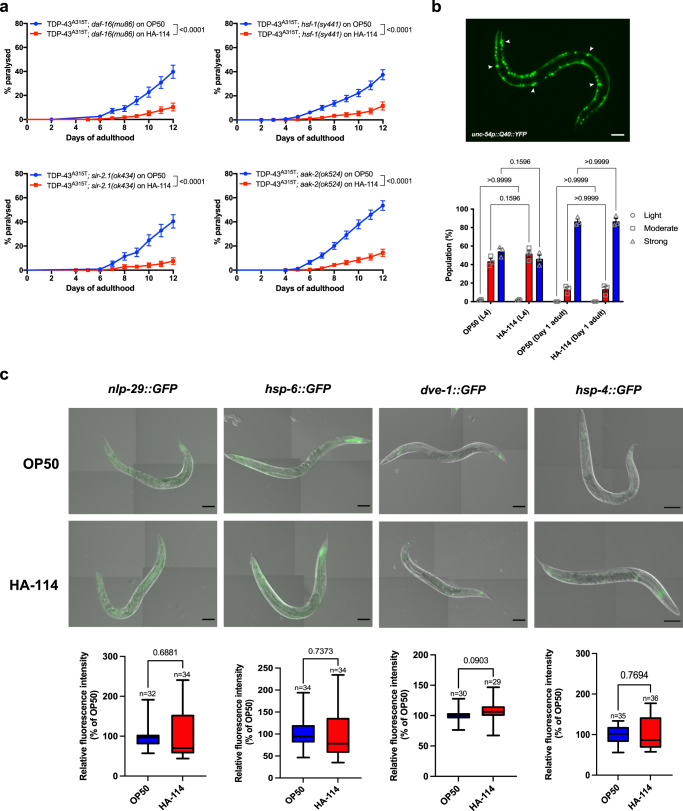
HA-114 does not require classic stress and metabolic pathways in *C. elegans* for neuroprotection. Neuroprotection provided by *L. rhamnosus* HA-114 was unaffected by **a** *daf-16(mu86)* deletion, nor by *hsf-1(sy441)* point mutation. Both *sir-2.1* and *aak-2* genes are not required for neuroprotection granted by HA-114 probiotics. **b** Aggregation was not altered by HA-114 in a polyQ model at L4 or at day 1 of adulthood. Scale bar = 100 μm. **c** HA-114 did not affect GFP expression of key genes of innate immune response (*nlp-29*), UPR^mito^ response (*hsp-6* and *dve-1*) or UPR^ER^ (*hsp-4*) Scale bar = 100 μm. For paralysis assays (**a**), curves were generated and compared using the log-rank (Mantel–Cox) test. TDP-43^A315T^;*daf-16(mu86)* on OP50 *n* = 248; TDP-43^A315T^;*daf-16(mu86)* on HA-114 *n* = 237; TDP-43^A315T^;*hsf-1(sy441)* on OP50 *n* = 279; TDP-43^A315T^;*hsf-1(sy441)* on HA-114 *n* = 237; TDP-43^A315T^;*sir-2.1(ok434)* on OP50 *n* = 118; TDP-43^A315T^;*sir-2.1(ok434)* on HA-114 *n* = 120; TDP-43^A315T^;*aak-2(ok524)* on OP50 *n* = 240; TDP-43^A315T^; *aak-2(ok524)* on HA-114 *n* = 239. For the aggregation assay (**b**), a two-way ANOVA was performed and for each conditions, *n* = 3 (with 237 worms evaluated over the 3 trials for OP50 L4, 236 worms for HA-114 L4, 230 worms for OP50 Day 1 adult and 216 worms for HA-114 Day 1 adult). For fluorescence quantification (**c**), an unpaired *t* test was performed and *n* are indicated in the figure. Data are presented as mean ± SEM. For boxplots, minimum, first quartile, median, third quartile, and maximum are shown.

Next, we sought to investigate the potential mechanisms underlying the neuroprotective effect of *L. rhamnosus* HA-114 by determining whether HA-114 required well-known and characterized pathways for exerting its neuroprotective effect. Using strains constructed with loss or partial loss-of-function mutations in worms with transgenic expression of the TDP-43^A315T^ mutant proteins, we disrupted fundamental metabolic, stress and signaling pathways in *C. elegans* to assess their role in mediating the neuroprotective effects of *L. rhamnosus* HA-114. We found that HA-114 remained able to rescue the paralysis phenotype in TDP-43^A315T^ animals independently of *daf-16/FOXO*^29^, *hsf-1/HSF1*^30^*, sir-2.1/SIRT1*^31^ and *aak-2/AMPk*^32^ (Fig. 2a). These results suggest that neither the insulin/IGF-1-mediated signaling pathway, the heat-shock response, the sirtuin pathway nor the AMP-activated protein kinase signaling pathway are required for the neuroprotection provided by HA-114. Similar results were obtained in our FUS^S57Δ^ worms (Supplementary Fig. 4).

Protein aggregation is considered as a major contributor to the etiology of neurodegenerative disorders and a hallmark of many late onset neurodegenerative diseases. Hence, we used transgenic *C. elegans* strains expressing YFP-tagged polyglutamine repeats (Q40, disease allele) under the *unc-54* promoter to assess the role of HA-114 on aggregation. These animals show progressive formation of Q40::YFP foci as they age^33^. HA-114 did not significantly decrease aggregation in L4 larvae, nor in day 1 adult worms when compared to worms fed with control OP50 bacteria (Fig. 2b). These results suggest that the effect mediated by HA-114 on disease-associated phenotypes occurs through an alternative pathway independent of aggregation. Subsequently, we screened several GFP reporter strains associated with genes implicated in major conserved stress response pathways. Transgenic reporter strains were fed for 24 h with OP50 or HA-114 and fluorescence was compared at day 1 of adulthood. Compared to worms fed with OP50, HA-114-fed worms showed no difference in GFP expression for the following reporters: *nlp-29::GFP*^34^, *hsp-6::GFP*^26^, *dve-1::GFP*^35^, *hsp-4::GFP*^36^*, hsp-60::GFP*^26^ *and hsp-16.2::GFP*^37,38^ (Fig. 2c and Supplementary Fig. 5). These results likely exclude the innate immune response (*nlp-29*), the mitochondrial unfolded protein response (UPR^mito^; *hsp-6*, *dve-1* and *hsp-60*), the endoplasmic reticulum unfolded protein response (UPR^ER^; *hsp-4*) and the cytoplasmic unfolded protein response (UPR^Cyt^; *hsp-16.2)* as key pathways activated by *L. rhamnosus* HA-114. Altogether, these results suggest that *L. rhamnosus* HA-114 neuroprotection may be independent of classic longevity and stress response pathways studied in *C. elegans*.

### Fatty acid metabolism genes are necessary for HA-114-mediated neuroprotection

**Fig. 3:**
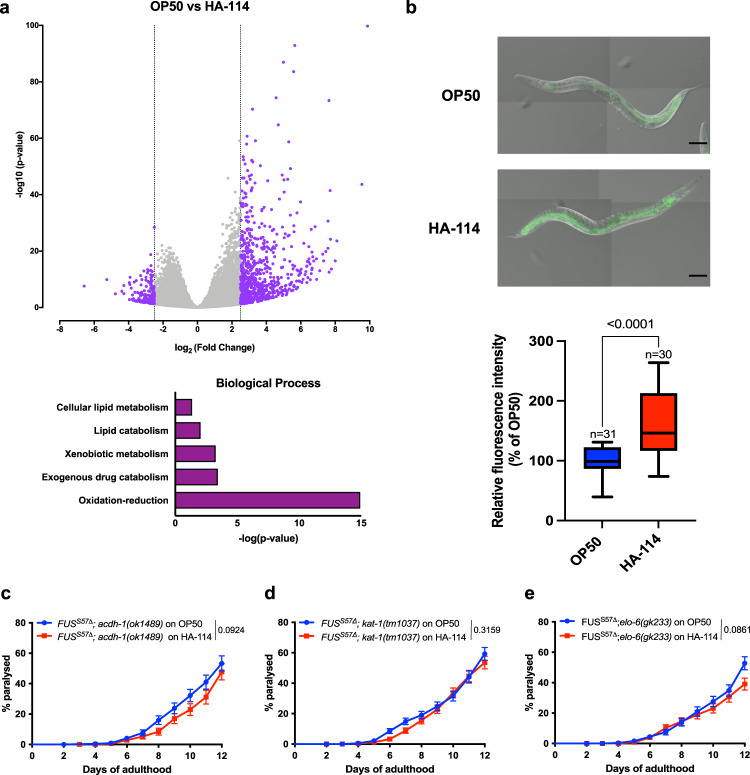
*acdh-1*, *kat-1* and *elo-6* are essential for neuroprotection provided by HA-114. **a** Volcano plot of RNA-Seq data of N2 worms fed with *Lacticaseibacillus rhamnosus* HA-114 and compared to worms fed with OP50. The data for all genes were plotted as log2 fold change versus -log10 of the adjusted *p* value. Gene ontology (GO) term analysis of the differentially overexpressed genes induced by HA-114 treatment. The GO term analysis was performed with PANTHER 11 by using *C. elegans* genes as background. Only the biological process terms with an enrichment of *p* value <0.01 are shown in this figure. Data: GEO accession: GSE189988; SRA study: SRP348888. **b** *L. rhamnosus* HA-114 significantly increase GFP expression in p*acdh-1*::GFP worms when compared to the same worms fed with OP50. Scale bar = 100 μm. Deletion of *acdh-1* (**c**)*, kat-1* (**d**), and *elo-6* (**e**) altered the beneficial effect of HA-114. For fluorescence quantification (**b**), an unpaired *t* test was performed and *n* are indicated in the figure. For paralysis assays (**c**–**e**), curves were generated and compared using the log-rank (Mantel–Cox) test. **c**: FUS^S57Δ^;*acdh-1(ok1489)* on OP50 *n* = 250; FUS^S57Δ^;*acdh-1(ok1489)* on HA-114 *n* = 190. **d**: FUS^S57Δ^;*kat-1(tm1037)* on OP50 *n* = 437; FUS^S57Δ^;*kat-1(tm1037)* on HA-114 *n* = 463. **e**: FUS^S57Δ^;*elo-6(gk233)* on OP50 *n* = 331; FUS^S57Δ^;*elo-6(gk233)* on HA-114 *n* = 285. For boxplots, minimum, first quartile, median, third quartile, and maximum are shown.

To gain insights on which category of genes might be implicated in HA-114’s protective activity, we conducted whole-worm coding RNA sequencing (RNA-seq) of N2 worms fed with different bacterial strains. We compared worms fed with *L. rhamnosus* HA-114 to those fed with OP50, considered as the standard laboratory food source of *C. elegans*, or *Bifidobacterium animalis subsp. lactis* B94, a strain with little effect on paralysis in our models of age-dependent neurodegeneration. RNA-seq results showed that the vast majority of differentially expressed genes were upregulated, with more than 300 genes having a fold change higher than 3 (Supplementary Tables 1 and 2 for OP50 vs HA-114 and Supplementary Tables 3 and 4 for OP50 vs B94). Enrichment gene ontology analysis showed that several categories of genes were upregulated in HA-114 samples, revealing lipid metabolism and oxidation-reduction as ones of the top categories (Fig. 3a and Supplementary Fig. 6). Many genes classified in the category oxidation-reduction are also involved in lipid metabolism processes. These results are consistent with recent studies suggesting that gut microbiome can influence lipid metabolism in various systems^39,40^. Moreover, lipid metabolism may play a role in both protection and deterioration of neurons in various neurodegenerative disorders^41,42^.

Next, we investigated the potential role of lipid metabolism and oxidation-reduction biological processes in the neuroprotection provided by HA-114. The *acdh-1* gene is the ortholog of human *ACADSB* and a member of the ACDH family, which has oxidoreductase activity and is involved in fatty acid β-oxidation^43^. We used a transgenic *acdh-1::GFP* reporter strain to study potential implication of *acdh-1* in HA-114’s neuroprotective effect^15^. We quantified GFP fluorescence intensity of day 1 adult worms fed with OP50 or *L. rhamnosus* HA-114. Transgenic *acdh-1::GFP* reporter strains displayed a significant change in fluorescence when fed with HA-114, although *acdh-1::GFP* worms displayed high GFP intensity when fed with control OP50 bacteria (Fig. 3b). Increased *acdh-1* is also observed at the mRNA levels in Day 1 FUS worms fed with HA-114 (Supplementary Fig. 7a). However, aged FUS worms (Day 6 and Day 9) fed with HA-114 showed decrease expression of *acdh-1* when compared to worms fed with OP50 (Supplementary Fig. 7b, c).

To confirm the role of *acdh-1* in HA-114-mediated neuroprotection, we generated a strain harboring the transgenic *FUS* allele in an *acdh-1* null background (FUS^S57Δ^;*acdh-1(ok1489)*)^15^. We observed no significant difference in paralysis rate over 12 days in these animals fed with either OP50 or HA-114 (Fig. 3c). However, we were unable to generate a similar strain using the TDP-43^A315T^ transgene. We then investigated the role of *acdh-10*, the ortholog of human *ACADM*, also involved in fatty acid β-oxidation. We generated FUS^S57Δ^; *acdh-10(syb1928)* worms, harboring a nonsense mutation in *acdh-10*. We found that HA-114 remained able to rescue the paralysis phenotype in our mutant *FUS* worms independently of *acdh-10/ACADM* (Supplementary Fig. 8). These results suggest that *acdh-1*, but not *acdh-10*, may be an essential gene for the neuroprotective effect provided by *L. rhamnosus* HA-114.

Extending our analysis to some other genes involved in fatty acid metabolism, we hypothesized that *kat-1*, which is an orthologue of human ACAT1 and also involved in fatty acid β-oxidation, and *elo-6*, an orthologue of human ELOVL3 and ELOVL6 coding for a fatty acid elongase, might be involved in the paralysis rescuing effect of HA-114^44,45^. Hence, we generated FUS^S57Δ^; *kat-1(tm1037)* and the FUS^S57Δ^; *elo-6(gk233)* strains, harboring a loss-of-function mutation in *kat-1* and *elo-6*. Feeding these mutant strains with HA-114 did not rescue paralysis phenotypes, suggesting a role for *kat-1* and *elo-6* in the neuroprotective effect of HA-114 (Fig. 3d, e). HA-114 also did not rescue paralysis in both TDP-43^A315T^; *kat-1(tm1037)* and TDP-43^A315T^; *elo-6(gk233)* strains, and even increased paralysis in *a elo-6* loss-of-function background (Supplementary Fig. 9). These three genes (*acdh-1, kat-1, elo-6*) share a common metabolic pathway, involving fatty acids elongation and degradation to provide energy to the cell (Supplementary Fig. 10). Collectively, these results suggest that fatty acid metabolism, and more specifically β-oxidation, might be involved in the mechanism underlying the neuroprotective effect of HA-114 in age-dependent neurodegeneration models.

### *L. rhamnosus* HA-114 fatty acids are essential for improving neurodegenerative phenotypes

**Fig. 4:**
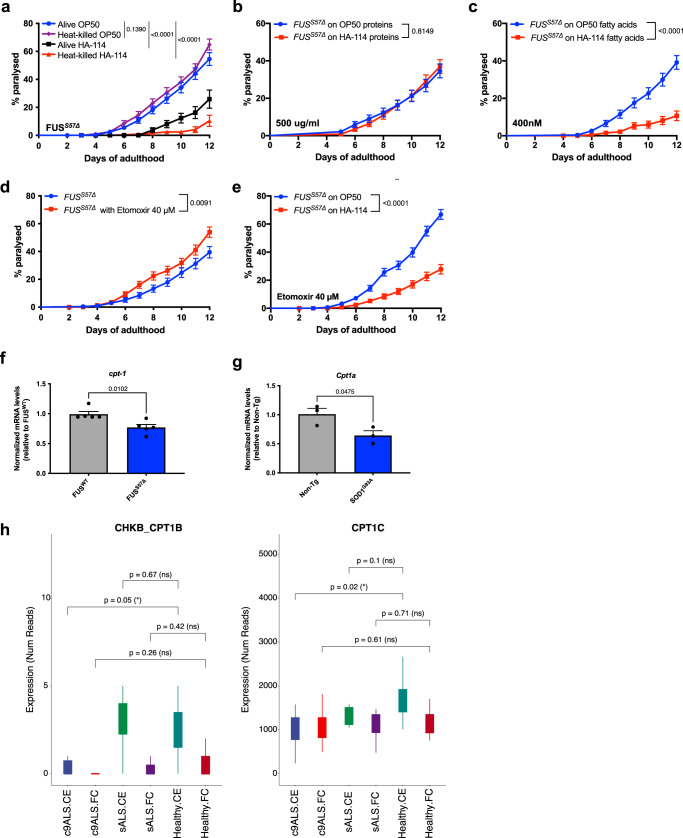
HA-114 fatty acids extracts, but not proteins, are sufficient to rescue paralysis. Transgenics were monitored from the adult stage, scored daily for paralysis and fed with control OP50 or HA-114’s individual components. **a** Mutant FUS worms fed with heat-killed *L. rhamnosus* HA-114 did not show paralysis phenotypes when compared to worms fed with OP50. **b** Protein extract from *L. rhamnosus* HA-114 failed to rescue age-dependent paralysis in ALS worm models. **c** When compared to fatty acid extract from OP50, fatty acids extract from HA-114 suppressed paralysis phenotypes in FUS animals. **d** 40 μM of Etomoxir, a *cpt-1/CPT1* inhibitor, increased paralysis in FUS transgenics. **e** The same concentration was not enough to block neuroprotective effects of *L. rhamnosus* HA-114, when compared to worms fed with OP50. **f** mRNA expression of *cpt-1* was significantly decreased in FUS^S57Δ^ worms when compared to FUS^WT^ animals. **g** mRNA expression of *CPT1A* was significantly decreased in livers of SOD1^G93A^ mice when compared to livers of Non-Tg animals. **h** Decrease expression of two transcripts related to carnitine palmitoyltransferase (CHKB_CPT1B and CPT1C) is observed in C9ORF72 patients when compared to controls. No significant change is observed in sporadic ALS. Data: GEO accession: GSM1642314; SRA study: SRP056477^60^. For paralysis assays (**a**–**e**) curves were generated and compared using the log-rank (Mantel–Cox) test. **a**: Alive OP50 *n* = 275; Heat-killed OP50 *n* = 212; Alive HA-114 *n* = 246; Heat-killed HA-114 *n* = 259. **b**: OP50 proteins *n* = 238; HA-114 proteins *n* = 240. **c**: OP50 fatty acids *n* = 240; HA-114 fatty acids *n* = 220. **d**: OP50 *n* = 220; Etomoxir *n* = 240. **e**: OP50 *n* = 326; HA-114 *n* = 302. For TaqMan assays (**f**, **g**), an unpaired *t* test was performed. **f**: *n* = 5 per condition. **g**: *n* = 3 per condition. For boxplots, minimum, first quartile, third quartile, and maximum are shown.

To better understand how HA-114 exerted its neuroprotective effect, we sought to investigate which component of *L. rhamnosus* HA-114 contributed to these beneficial effects. Recently, differential effects between live and heat-killed probiotics on general health have been observed^46,47^. To investigate the importance of HA-114 viability for its rescue effect on the disease phenotype, we fed the FUS^S57Δ^ worms with live or heat-killed bacteria from day 1 to day 12 of adulthood. We observed a similar protective effect against paralysis in animals fed with either heat-killed or live HA-114 compared with animals fed with OP50 (Fig. 4a). To identify which component of HA-114 was responsible for its neuroprotective activity, we assessed whether protein/amino acid or fatty acid (FA) extracts from HA-114, mixed with standard OP50 and used as a food source, were sufficient to provide beneficial effects against the age-dependent paralysis phenotype in the ALS model strain FUS^S57Δ^ worms. While FUS^S57Δ^ worms fed with 500 µg/ml protein extract from either OP50 or HA-114 displayed a similar paralysis phenotype (Fig. 4b), those fed with 400 nM of FA extract from HA-114 showed a reduction of the phenotype compared to controls (Fig. 4c).

Interestingly, fatty acid extract from HA-114 was able to increase GFP signals in the transgenic *acdh-1::GFP* reporter strain, yet to a lesser extent than HA-114 (Supplementary Fig. 11). It has been previously shown that the short-chain fatty acid propionate activates the *acdh-1* promoter^18,48^. In order to assess if activation of *acdh-1* through propionate supplementation was enough to rescue paralysis phenotype in our FUS^S57Δ^ worms, we fed our worms with two concentration of propionate, either 400 nM (concentration used for fatty acid extract) or 100 μM (average concentration of propionate produced by *L. rhamnosus* strains^49^). Propionate failed to rescue the paralysis phenotype in FUS^S57Δ^ worms, while having no deleterious effect in addition with HA-114, showing that large concentrations of propionate had no effect in our FUS worms (Supplementary Fig. 12a). While propionate is known to increase *acdh-1::GFP* expression, previous publications reported that vitamin B12 has the ability to inhibit the *acdh-1::GFP* signal^18,48,50^. Hence, we used vitamin B12 as an inhibitor of *acdh-1* in our FUS model and evaluated paralysis rates after the treatment. Interestingly, addition of vitamin B12 in combination with our HA-114 strain prevented the neuroprotective action of the probiotics (Supplementary Fig. 12b), confirming our previous results showing that *acdh-1* is essential in HA-114’s mechanism of action (Fig. 3b, c). Taken together these data suggest that *acdh-1* activation plays an important role in HA-114 neuroprotection and that this activation is independent of short-chain fatty acids like propionate.

The fatty acid extract used in this study contains a mix of FA from HA-114, including medium-chain fatty acids (MCFAs, 6-12 carbons long), long-chain fatty acids (LCFAs, 13-21 carbons) and very-long chain fatty acids (VLCFAs, 22 carbons and more). Contrary to short or medium-chain fatty acids which can freely enter the mitochondria to be oxidized, long-chain fatty acids need to be actively transported into the mitochondria to be part of the β-oxidation process. The classical transporter for LCFAs is the carnitine-shuttle, a conserved protein complex located on the mitochondrial membrane^43^. Specifically, carnitine palmitoyltransferase-1 (*cpt-1*/CPT1) is an important component of the carnitine shuttle complex at the outer face of the mitochondrial membrane. To assess the role of the carnitine shuttle on the motor phenotype of FUS^S57Δ^ animals, we used etomoxir, a specific CPT-1 inhibitor that prevents LCFA transport into the mitochondria by blocking the formation of long chain acylcarnitines, key components in the carnitine-shuttle machinery. We hypothesized that if LCFAs were mediating the rescue effect provided by HA-114, inhibiting LCFA transport should exacerbate the neurodegeneration phenotype in the FUS^S57Δ^ *C. elegans* model by impairing proper β-oxidation and altering energy homeostasis. As expected, we observed that blocking *cpt-1/*CPT1 was sufficient to significantly increase paralysis phenotypes in our model (Fig. 4d). Furthermore, treating OP50- or HA-114-fed FUS^S57Δ^ worms with 40 μM of Etomoxir revealed that blocking *cpt-1* was not sufficient to prevent the HA-114 bacterial strain from rescuing the paralysis phenotype (Fig. 4e). Etomoxir is known to cause oxidative stress and off-target effects at high doses (>100 μM)^51,52^. In order to assess whether Etomoxir can contribute to oxidative stress in our experimental set-up, we tested two Etomoxir concentrations: 10 uM, known to effectively block FA β-oxidation^52^ and 40 μM, used in our study and other *C. elegans* publications^53–55^. We examined if Etomoxir was able to increase *hsp-6::GFP* expression after 24 h exposure. Both concentrations did not increase *hsp-6::GFP* expression when compared to worms fed with OP50 (Supplementary Fig. 13). Collectively, these results support the notion that impaired β-oxidation can worsen motor phenotypes and that HA-114-derived fatty acids may bypass the carnitine-shuttle to enter the mitochondria to be processed by the β-oxidation chain. These findings are consistent with evidence of an alternative pathway implicating other transporters for LCFAs, FAT/CD36 and SLC27^56–58^.

To investigate the potential role of the carnitine shuttle in ALS pathogenesis, we then assessed *cpt-1* expression levels in FUS^S57Δ^ and FUS^WT^ transgenic animals. Interestingly, qRT-PCR using TAQMan probes of *cpt-1* revealed that mutant FUS animals expressed less *cpt-1* transcripts when normalized to *ama-1* mRNA level and compared to control animals (Fig. 4f). CPT1A mRNA expression is also decreased in liver of hSOD1^G93A^ transgenic mice, a well-characterized mouse model of ALS^59^, when compared to non-transgenic (Non-tg) littermates (Fig. 4g). Finally, bioinformatics analysis of brain transcriptome from Prudencio et al. dataset revealed that expression of two transcripts related to carnitine palmitoyltransferase (CHKB_CPT1B and CPT1C) are decreased in cerebellum, but not in frontal cortex, of *C9orf72* patients^60^ (Fig. 4h). Expression of other genes implicated in β-oxidation and lipid metabolism are also differentially expressed in the cerebellum and frontal cortex of *C9orf72* patients (Supplementary Fig. 14). Taken together, these results suggest that fatty acids are the active component of HA-114 bacterial strain and they can bypass the carnitine shuttle machinery. Moreover, these results point toward intrinsic carnitine shuttle issues in ALS pathogenesis.

### *L. rhamnosus* HA-114 restores lipid homeostasis in age-related neurodegeneration models

**Fig. 5:**
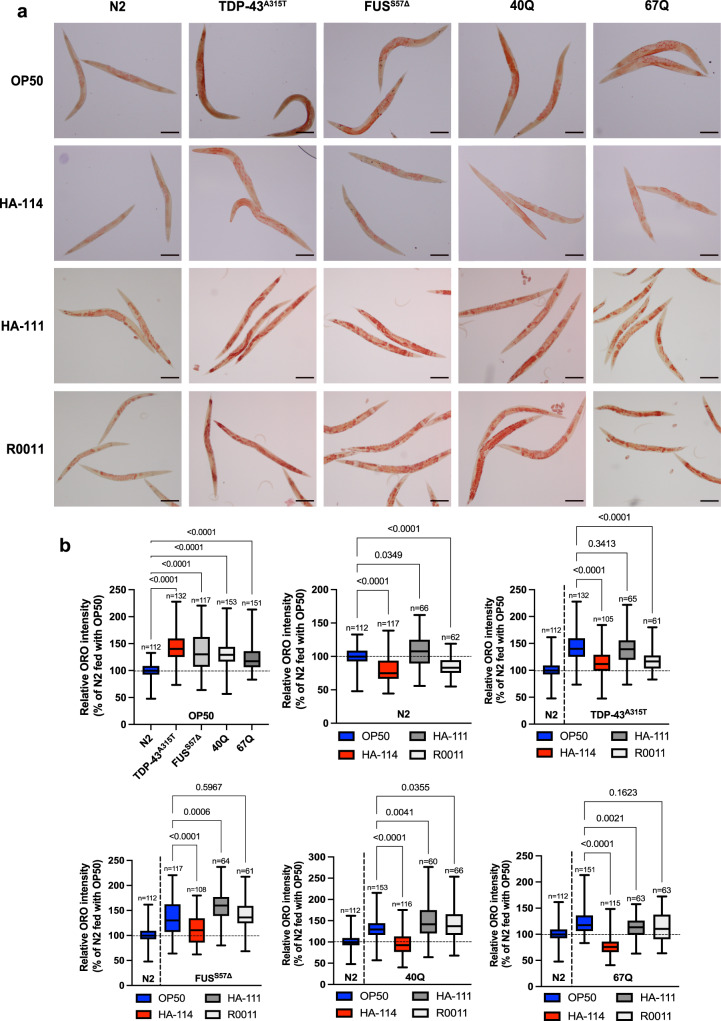
*Lacticaseibacillus rhamnosus* HA-114 modulate lipid accumulation in worm age-dependent neurodegeneration models. **a** Representative images of worms fed with OP50, *L. rhamnosus* HA-114, *L. rhamnosus* HA-111 or *L. rhamnosus* R0011 and stained with Oil Red O. N2 worms show a basal level of lipid accumulation. **b** Quantification of Oil Red O show increased fat accumulation in worms expressing FUS^S57Δ^, TDP-43^A315T^, and polyQ expansion (40Q and 67Q) when compared to N2. Worms fed with HA-114 showed a significant decrease of lipid accumulation, while both HA-111 and R0011 *rhamnosus* strains failed to decrease fat accumulation in all models. For the Oil Red O quantification graphs (**b**), Brown–Forsythe and Welch ANOVA were performed and *n* are indicated in the figure. Scale bar = 100 μm. For boxplots, minimum, first quartile, median, third quartile, and maximum are shown.

Over the last few years, many studies have implicated impaired lipid metabolism in various neurodegenerative disorders, including Alzheimer’s disease and ALS^42,61,62^. Since our data suggested that HA-114 requires the fatty acid metabolism key component *kat-1* and increases *acdh-1* expression, we hypothesized that the age-related neurodegeneration models we used might have disrupted lipid equilibrium and that HA-114 might restore lipid content to normal levels. We stained day 1 adult worms with Oil-Red-O dye to visualize neutral lipids. We observed higher content of lipid droplets in animals expressing TDP-43^A315T^, FUS^S57Δ^, Q40 or Q67 transgenes when compared to N2 animals (Fig. 5a, b). Interestingly, this lipid accumulation was restored to control levels when animals were fed with HA-114 instead of OP50.

HA-114 was the only *L. rhamnosus* strain to restore lipid homeostasis in all age-related neurodegeneration models, while *L. rhamnosus* HA-111 increased lipid accumulation in several worm strains, including N2, FUS^S57Δ^, Q40 and Q67. We then wanted to investigate if HA-114 fatty acids also had the ability to restore lipid homeostasis since we demonstrated that they act as the active component of HA-114’s neuroprotective effect. Hence, we stained with Oil-Red-O dye 1 adult worms fed with either OP50, HA-114, OP50 fatty acids or HA-114 fatty acids. We observed that worms fed with HA-114 fatty acids, but not OP50 fatty acids, had lower levels of lipid accumulation in animals expressing TDP-43^A315T^, FUS^S57Δ^, Q40 and Q67 transgenes (Supplementary Fig. 15a, b). However, this effect was not observed in N2 animals. Our data demonstrate that impaired lipid homeostasis is a feature of various models of neurodegenerative disorder and that HA-114 can rescue this phenotype. Lipid accumulation is often associated with impaired β-oxidation^63^, which is consistent with what we have observed in our models.

### Impaired β-oxidation shortens lifespan and exacerbates neurodegeneration

**Fig. 6:**
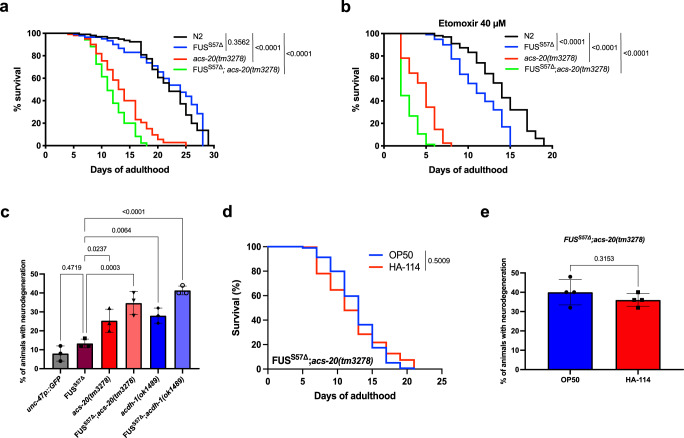
Impaired β-oxidation contributes to neurodegeneration and shortens lifespan. Worms were monitored from the adult stage until death. **a** *acs-20(tm3278)* and FUS^S57Δ^;*acs-20(tm3278)* worms have shorter lifespan than N2 and FUS^S57Δ^; animals. **b** Etomoxir (40 μM), a *cpt-1/CPT1* inhibitor, shorten lifespan of FUS^S57Δ^, *acs-20(tm3278)* and FUS^S57Δ^;*acs-20(tm3278)* worms when compared to N2. **c** *acs-20(tm3278)*, FUS^S57Δ^;*acs-20(tm3278)*, *acdh-1(ok1489)* and FUS^S57Δ^;*acdh-1(ok1489)* animals have a higher rate of neurodegeneration compared to transgenic GFP controls at day 1. **d** HA-114 does not extend lifespan in FUS^S57Δ^;*acs-20(tm3278)* animals nor **e** decreases neurodegeneration phenotype at day 1 of adulthood. For lifespan assays (**a**, **b**, **d**), curves were generated and compared using the log-rank (Mantel–Cox) test. **a**: N2 *n* = 106; FUS^S57Δ^ *n* = 104; *acs-20(tm3278)* *n* = 104; FUS^S57Δ^;*acs-20(tm3278)* *n* = 105. **b**: N2 *n* = 107; FUS^S57Δ^ *n* = 105; *acs-20(tm3278)* *n* = 105; FUS^S57Δ^;*acs-20(tm3278)* *n* = 105. **d**: OP50 *n* = 210; HA-114 *n* = 212. For neurodegeneration assays (**c**, **e**), one-way ANOVA (**c**) and unpaired *t* test (**e**) were performed. For **c**, each condition *n* = 3 (25 worms per *n*). For **c**, each condition *n* = 4 (25 worms per *n*). Data are presented as mean ± SEM.

As previously mentioned, our findings are consistent with evidence of an alternative pathway to the carnitine shuttle implicating other transporters for LCFAs, including FAT/CD36 and SLC27 family members. To further investigate the potential implication of this pathway in disease phenotypes and neurodegeneration, we generated a strain harboring the transgenic FUS allele with an *acs-20* null background, the worm ortholog of *SCL27A*^64^. Since *acs-20(tm3278*) mutants display impaired locomotion at a young age, we were unable to study paralysis phenotypes on this strain nor in *FUS*^*S57Δ*^*;acs-20(tm3278)* worms. To study other phenotypes, we evaluated lifespan of these animals. We hypothesized that *acs-20* mutants might negatively regulate longevity and that our mutant FUS animals might be sensitive to this loss-of-function. Interestingly, while FUS^S57Δ^ transgenics do have lifespan like N2, *acs-20(tm3278)* exhibited shortened lifespan that was exacerbated in our *FUS*^*S57Δ*^*;acs-20(tm3278)* animals (Fig. 6a). To assess the effects of complete inhibition of LCFAs transport, we evaluated lifespan of our worms using Etomoxir, a specific inhibitor of *cpt-1/CPT1*. We observed that blocking both *cpt-1/*CPT1 and *acs-20/SLC27A* was sufficient to significantly decrease lifespan in our FUS^S57Δ^ animals compared to N2. Moreover, *acs-20(tm3278)* mutants had shorter lifespan. This effect was also additive in our *FUS*^*S57Δ*^*;acs-20(tm3278)* animals, which displayed the shortest lifespan of all conditions (Fig. 6b). Next, we assessed the contributions of *acs-20(tm3278)*, or *acdh-1(ok1489)* mutants to neurodegeneration. While N2 and mutant FUS display similarly low rates of neurodegeneration at a young age, both *acs-20* and *acdh-1* mutants were associated with GABAergic motor neuron loss as early as day 1. We also found a significant increase in motor neuron degeneration levels in both *FUS*^*S57Δ*^*;acs-20(tm3278)* and *FUS*^*S57Δ*^*;acdh-1(ok1489)* animals (Fig. 6c). We then assessed if HA-114 could affect lifespan or neuronal health in our *FUS*^*S57Δ*^*;acs-20(tm3278)* animals. We observed that HA-114 treatment failed to extend lifespan and to prevent GABAergic degeneration at day 1 of adulthood in *FUS*^*S57Δ*^*;acs-20(tm3278)* worms (Fig. 6d, e). Taken together, these results suggest that *acdh-1/ACADSB* and *acs-20/SLC27A* can influence lifespan, and that impaired β-oxidation might play an important role in neurodegeneration.

### *L. rhamnosus* HA-114 displays a unique lipid profile

**Fig. 7:**
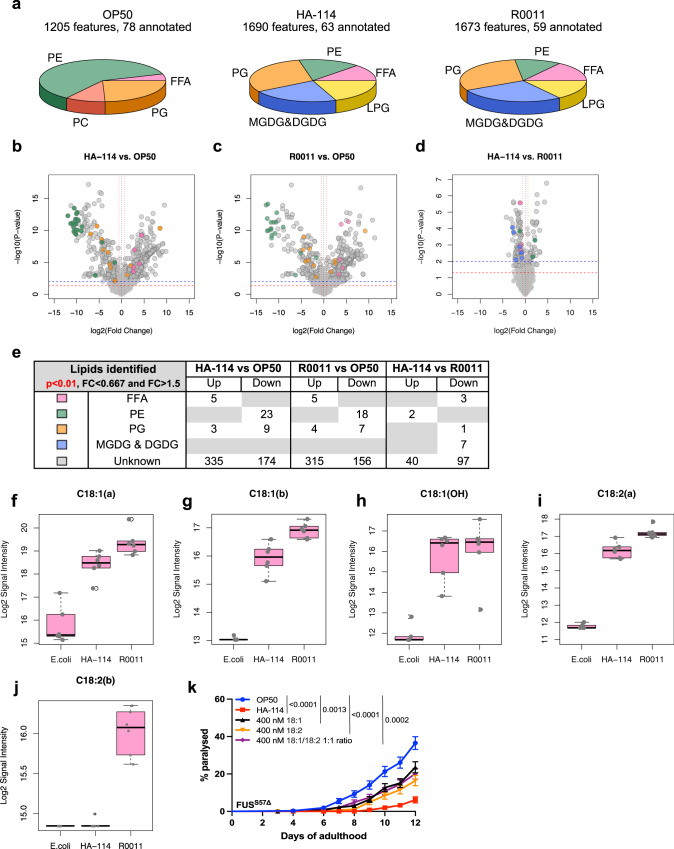
Lipidomics analysis reveal HA-114 unique lipidic profile. **a** Lipid composition (pie charts) of OP50, *L. rhamnosus* HA-114 and *L. rhamnosus* R0011 based on the presence of the annotated lipids in at least 80% of the samples. **b**–**d** Volcano plots from LC-QTOF-based untargeted lipidomic. Red line represents: *p* value = 0.05 and blue line: *p* value = 0.01. Vertical red lines: Fold change >1.5 or <0.667. Dot color represents the subclass of the lipid annotated. **b** Volcano plot from HA-114 compared to *E. coli* depicting 1068 MS features including 549 that meet the threshold of significance and fold change. **c** Volcano plot from R0011 compared to E. coli depicting 1046 MS features including 505 that meet the threshold of significance and fold change. **d** Volcano plot from HA-114 compared to R0011 depicting 1761 MS features including 150 that meet the threshold of significance and fold change. **e** Table listing the annotated lipids that were found to be up- or down-regulated for each comparisons meeting the criteria of significance and fold change, with their color symbols. **f** C18:1 isomer a (HA-114 vs OP50 *p* = 6.0805968e-05; R0011 vs OP50 *p* = 3.619222e-06; HA-114 vs R0011 *p* = 0.0130286415), **g** C18:1 isomer b (HA-114 vs OP50 *p* = 1.110920617e-07; R0011 vs OP50 *p* = 1.08875064e-11; HA-114 vs R0011 *p* = 0.00214720654), **h** C18:1(OH) hydroxy oleic acid (HA-114 vs OP50 *p* = 1.7197553827e-05; R0011 vs OP50 *p* = 7.3305759946e-05; HA-114 vs R0011 *p* = 0.77912583), **i** C18:2 isomer a (HA-114 vs OP50 *p* = 5.9513787e-10; R0011 vs OP50 *p* = 4.1309010e-12; HA-114 vs R0011 *p* = 0.001071952), and **j** C18:2 isomer b (For HA-114 vs OP50 and R0011 vs OP50: no p-value, C18:2 n-3 was absent from OP50 samples; HA-114 vs R0011 *p* = 2.702653227e-06). **k** Transgenics were monitored from the adult stage, scored daily for paralysis. Mutant FUS worms fed with oleic acid, linoleic acid and a mix of both fatty acid (1:1 ratio) showed decrease paralysis phenotype when compared with worms fed with OP50. For lipidomics analysis, *n* = 6 for each bacterial strain. Raw data and untargeted lipidomic results analysis are available in Supplementary Data files 1 and 2. For paralysis assays (**k**), curves were generated and compared using the log-rank (Mantel–Cox) test. **h**: OP50 *n* = 314, HA-114 *n* = 312, 400 nM C18:1 *n* = 317, 400 nM C18:2 *n* = 310, 400 nM C18:1/C18:2 1:1 ratio *n* = 318. FFA free fatty acids, PE diacylglycerophosphoethanolamines, PC diacylglycerophosphocholines, PG diacylglycerophosphoglycerols, MGDG monoglycosyldiacylglycerols, DGDG diglycosyldiacylglycerols, LPG lysl-diacylglycerophosphoglycerols. For boxplots, minimum, first quartile, median, third quartile, maximum, and each data point are shown.

To find which lipids, present in *L. rhamnosus* HA-114 might play a key role in the neuroprotective effect observed, we performed LC-QTOF-based untargeted lipidomic on three bacterial strains: OP50, *L. rhamnosus* HA-114 and *L. rhamnosus* R0011. Lipid composition of each strain was determined based on the presence of the annotated lipids in at least 80% of 6 replicate samples. OP50 displayed a very different lipid profile than both *L. rhamnosus* strains, with a high proportion of diacylglycerophosphoethanolamines (PE) and no monoglycosyldiacylglycerols (MGDG) and diglycosyldiacylglycerols (DGDG) (Fig. 7a). Lipid profiles were then compared and a threshold of significance of *p* < 0.01 in combination with a fold change higher than 1.5 or lower than 0.67 was selected for further analysis. The comparison between HA-114 and OP50 identified 1068 MS features including 549 that met our analysis criteria (Fig. 7b), while the comparison of R0011 and OP50 depicted 1046 MS features including 505 that met our analysis criteria (Fig. 7c). Of most interest, comparison between HA-114 and R0011 identified 1761 MS features including 150 that meet the threshold of significance and fold change (Fig. 7d). Several lipids were differentially expressed between the three bacterial strains, with a high proportion of yet unknown lipids (Fig. 7e). Because our previous results showed that fatty acids are essential for HA-114’s neuroprotective effect, the following work was focused on free fatty acids annotated in OP50, HA-114 and R0011.

While myristic acid (C14:0), hydroxypalmitic acid (C16:0(OH)), and stearic acid (C18:0) showed similar levels in the three bacterial strains (Supplementary Fig. 16a, c, d), palmitic acid (C16:0) was downregulated in samples of both HA-114 and R0011 strains when compared to OP50 (Supplementary Fig. 15b). Interestingly, two isomers of C18:1 (a and b), one isomer of C18:2 (a) and hydroxyl oleic acid (C18:1(OH)) were all upregulated in both HA-114 and R0011 (Fig. 7f–i). Although we were not able to distinguish between n-7 and n-9 for C18:1 (isomer a and b, Fig. 7f, g) and between n-3 and n-6 for C18:2 (isomer a and b, Fig. 7h–j), we decided to focus on oleic acid and/or linoleic acid for our last experiment since both have been associated with neuroprotective effects^65,66^. We then tested in our FUS^S57Δ^ worms fatty acid upregulated in both *L. rhamnosus* strains but at different degrees of intensity: C18:1 and C18:2. Worms fed with either 400 nM of oleic acid or linoleic acid showed decreased paralysis when compared to worms fed with OP50 (Fig. 7k). Similarly, worms fed with a blend of oleic acid/linoleic acid 1:1 showed similar results. However, none of the lipids rescued paralysis at the same levels than HA-114 alone. Collectively, these results indicate that *L. rhamnosus* HA-114 has a distinct lipid profile but further characterization of this profile is needed, especially outside of the FFA profile.

## Discussion

Microbiome research has highlighted the importance of the gut–brain axis in human health. Gut flora has primarily been studied in cases of inflammatory diseases, but emerging data is beginning to link microbiome components to neurodegenerative disorders, including Parkinson’s and Alzheimer’s diseases. Although some bacterial strains have been suggested to play a role in neurodegeneration, so far very few strain has been formally associated with this process^2,4–7^.

We investigated the potential beneficial role of microbiome and dietary supplementation in neurodegenerative diseases and discovered that a probiotic strain, *L. rhamnosus* HA-114, was able to positively modulate disease phenotypes in multiple *C. elegans* models of age-dependent neurodegeneration. Our results demonstrate the ability of a bacterial strain to restore multiple motor or neurodegeneration phenotypes. HA-114 is the only *L. rhamnosus* strain tested showing neuroprotective effects. A recent study showed that HA-114 improved hippocampal dependent cognition deficits in a rodent model of Parkinson’s disease^67^. We excluded the contribution of classic metabolic and stress responses pathways into the beneficial effect provided by HA-114 in our models.

Our investigation of gene expression signatures of HA-114 pointed towards lipid metabolism as perhaps a key mechanism linked to neuroprotection. However, we acknowledge that a more comprehensive investigation with the inclusion of additional bacterial strains, including *L. rhamnosus* strains other than HA-114 will be required to identify strain specific gene expression profiles.

However, we also identified *acdh-1/ACADSB*, *kat-1/ACAT1* and *elo-6/ELOVL3/6* as key components of the neuroprotection provided by the HA-114 strain. Two of those genes are involved in two distinct metabolic pathways: fatty acid metabolism, more precisely mitochondrial β-oxidation, and branch-chained amino acid breakdown (BCAA). Our results show that providing protein extract from HA-114, was not sufficient to recapitulate the beneficial effect seen with HA-114. Interestingly, HA-114-derived fatty acids were sufficient to rescue motor phenotype in our *C. elegans* ALS model. These results suggest that β-oxidation is the favored pathway in this context. Mitochondrial fatty acid β-oxidation disorders are associated with many symptoms including neuropathy^68^. Moreover, we identified lipid homeostasis dysregulation in various *C. elegans* models of age-dependent neurodegeneration. Augmentation of lipid droplets is often associated with improper β-oxidation, reflecting the incapacity of mitochondria to oxidized fatty acids and use them as an energy source^69^. Finally, we identified *acs-20/SLC27A*, an alternative entry point for LCFAs into the mitochondria, as an essential modulator of lifespan in our *C. elegans* ALS model as well as an important contributor to neurodegeneration. However, we acknowledge that a more comprehensive investigation of β-oxidation is required to have the full mechanistic scope of HA-114’s neuroprotective effect. Further work is ongoing to achieve this goal.

**Fig. 8:**
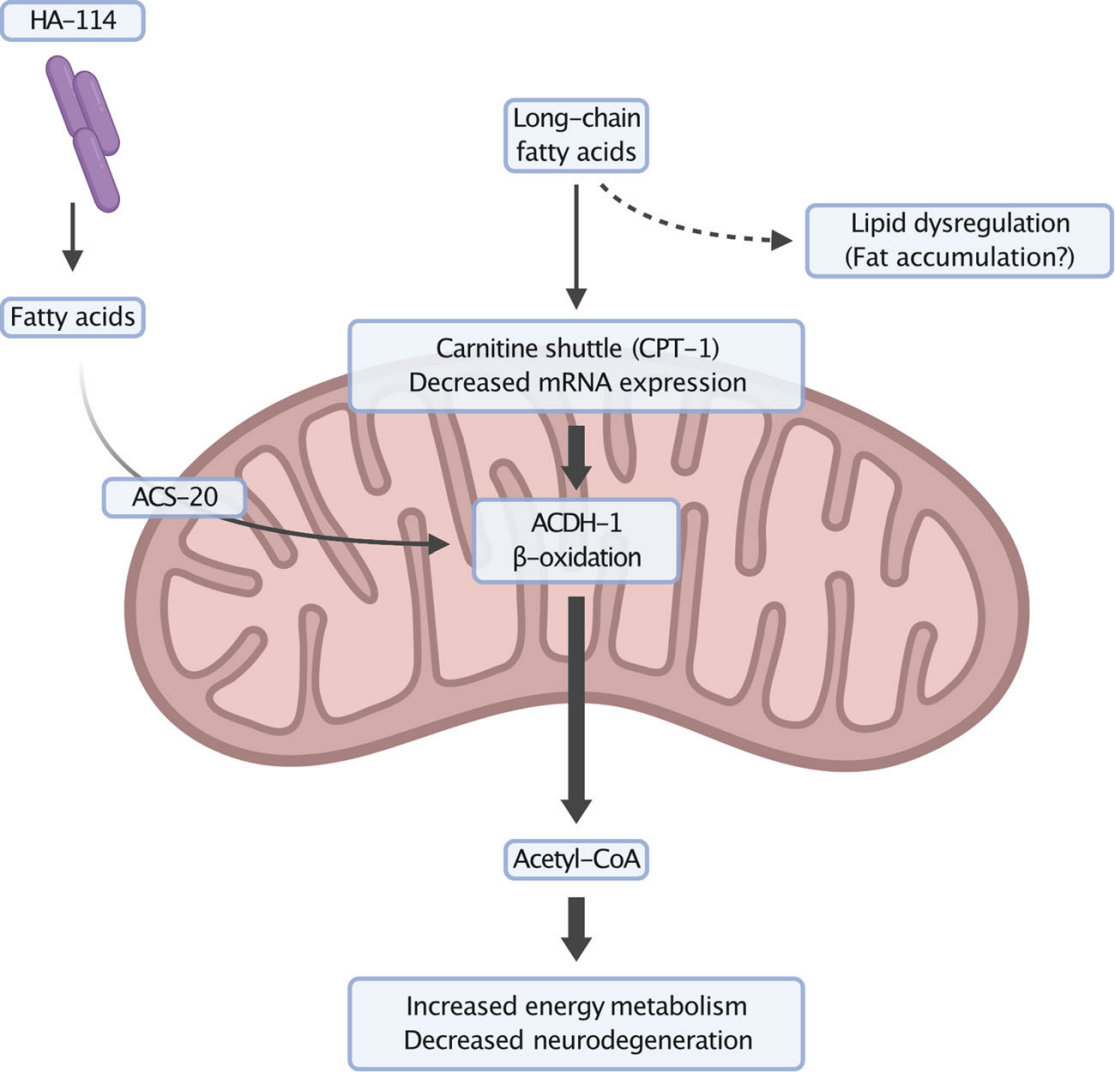
Neuroprotection mechanism of *Lacticaseibacillus rhamnosus* HA-114. ALS models have impaired carnitine shuttle, a mechanism to transport long chain fatty acids across the mitochondrial membrane for energy production via β-oxidation. Fatty acids, supplied by the probiotic bacteria, is believed to enter the mitochondria independently of the carnitine shuttle to participate in a few rounds of β-oxidation helping to stabilize energy metabolism, resulting in decreased neurodegeneration and improved lipid homeostasis. Created with BioRender.com.

An important feature of many age-related neurodegenerative disorders is the accumulation and aggregation of misfolded proteins in the cytosol suggesting potential common pathogenic mechanisms. We demonstrated that *L. rhamnosus* HA-114 was not able to prevent aggregation, while still being effective to protect neurons from degeneration. Neurodegeneration may in part be caused by metabolic and energy imbalances associated with the expression of mutant genes in aging neurons. Interventions that can compensate for the loss of energy production may bolster the cell’s ability to restore lipid homeostasis and energy production, ultimately delaying or halting neurodegeneration. Our results suggest that dietary supplementation with *L. rhamnosus* HA-114 provides key nutrients driving energy production, helping to mitigate metabolic dysfunction leading to neurodegeneration (Fig. 8).

*C. elegans* have been recently used as a model to study dietary supplementation of probiotics and their effect on neurodegeneration. The model organism helped identified *Bacillus subtilis* as neuroprotective in *C. elegans* models of Alzheimer’s disease and against α-synuclein aggregation^70,71^. Interestingly, *Bacillus subtilis’* mechanism behind protection against α-synuclein aggregation is *daf-16* dependent and involves sphingolipid metabolism^71^. Other neuroprotective bacterial strain have been linked to *daf-16*, including *E*. *coli* HT115 and *L. rhamnosus* CNCM I-3690^72,73^. Our study demonstrates that the neuroprotective effects of gut microbiome modulation through dietary intervention of probiotics using several neurodegeneration models may follow a mechanism independent of *daf-16*.

Disrupted energy homeostasis is well studied in ALS, as well as in other neurodegenerative disorders^74^. The majority of ALS patients present hypermetabolism, hyperlipidemia as well as insulin resistance^75^. Interestingly, ALS patients with high body mass index (BMI) have better prognosis than patient with BMI within the normal values. Moreover, obese individuals are less likely to develop ALS than individuals with average weight^76^. However, BMI does not differentiate between fat or lean body mass. Furthermore, ALS patients display non-alcoholic fatty liver disease, without being overweight or obese, suggesting lipid homeostasis impairment^41,77^. Increased lipid synthesis and accumulation in WT mice lead to ALS phenotypes, including muscular atrophy, neurodegeneration and paralysis^78^. Similar lipid disequilibrium phenotypes can be observed in transgenic mouse models of ALS^79^ and Alzheimer’s disease^80^. Hepatic steatosis (fatty liver degeneration) seems to be a common phenotype associated with neurodegenerative disorders and neurodegeneration caused by viral infections^81–83^. Interestingly, untargeted lipidomics done in plasma from ALS patients identified significant changes in pathways involved in energy metabolism, including fatty acid metabolism^84^. Other publications also reported lipid changes and altered lipid metabolism in several models of ALS and identified these changes as potential contributors to the disease^78,85–89^. Finally, histone deacetylase (HDAC) inhibitors, recently investigated as a potential therapeutics in ALS, has been shown to mitigate lipid metabolism alterations in the spinal cord of symptomatic FUS mice^88,90^. A common clinical observation in ALS patients is weight loss. Although fat accumulation is a phenotype we see in *C. elegans* models, it is not possible to directly extrapolate this phenomenon to ALS patients. However, it is a marker of impaired lipid homeostasis. Even if *C. elegans* are devoid of specialized adipose tissue or liver, many of the genes required for lipid metabolism are highly conserved.

We demonstrated that the carnitine shuttle is impaired in animals expressing mutant ALS genes, with reduced mRNA expression of *cpt-1/CPT1*. ALS patients are known to have lower L-carnitine levels than healthy subjects^91–93^ and lower serum level of L-carnitine is associated with higher severity of the disease^93^. L-carnitine is essential for β-oxidation via the carnitine shuttle, where CPT1/2 are required. This shuttle is typically used for long or very long-chain fatty acids, while short and medium chain fatty acids can enter the mitochondria independently. However, if ALS patients have lower L-carnitine, it means they have decreased β-oxidation. Our data suggest that the real problem may not be the low L-carnitine levels by itself, but that ALS patients/models have less active CPT1/2 receptor activity. This could also explain why ALS patients have higher long-chain fatty acids in their cytosolic triacylglycerol pools^94^, and these molecules may contribute to loss of lipid homeostasis if not properly metabolized in the mitochondria.

Interestingly, L-carnitine treatment was protective in a mouse model of ALS^95^, and promising endpoints were observed in a small ALS clinical trial for patients treated with acetyl-L-carnitine^96^. Data from Drosophila ALS models points toward dysfunction of the carnitine shuttle as a potential mechanism^97^. Altogether, these studies suggest that impaired β-oxidation, and perhaps the carnitine shuttle contributes to motor neuron degeneration in ALS. Since misfolded proteins can interact with mitochondria^98–100^, they may directly interact with carnitine shuttle proteins, or alter mitochondrial morphology leading to impairment of the shuttle. A recently published study showed that downregulation of CPT1 activity in SOD1^G93A^ mice resulted in amelioration of disease symptoms and shifted the gut microbiome communities towards a protective phenotype in these animals^101^. Interestingly, the authors also showed that upregulation by high-fat diet resulted in a more aggressive disease progression^101^. However, various studies demonstrated that a high-fat diet and ketogenesis have the ability to prevent motor neuron loss and delay symptoms in SOD1^G93A^ mice^102–105^, while enhancement of carnitine shuttle metabolism have proved to be effective as well^95,96^. Further investigations are needed to fully understand the role of CPT1 in ALS pathogenesis.

Our results also demonstrate that loss-of-function of both *acs-20/SLC27A* and *acdh-1/ACADSB* have the capacity to induce motor neuron loss in a model prone to neurodegeneration. Finally, *ACSL5*, a gene directly implicated in lipid metabolism has been recently linked to ALS^106,107^. Our results show differential expression of *ACSL5* in both frontal cortex and cerebellum in a cohort of *C9orf72* patients (Supplementary Fig. 13c). In this study we demonstrate that gut microbiome modulation via probiotic intervention is protective in several age-related neurodegeneration models, and there may be a link between impaired β-oxidation and neurodegeneration.

ALS drives more changes than the ones affecting the CNS, even before disease onset, with studies showing disruption in a wide range of metabolic pathways, including glucose^102,108–118^, lipids^41,77,119–129^, and mitochondrial networks and bioenergetics^130–143^. These modifications have been identified among neurons, but also outside the nervous system. Most genes associated with ALS are ubiquitously expressed and are not exclusively associated with neurons. Despite the vulnerability of motor neurons to these mutations, it is reasonable to hypothesize that they might impact other systems. However, there is a lack of research investigating the involvement of systems other than the nervous system in ALS pathogenesis.

Our transgenic ALS *C. elegans* models express FUS and TDP-43 only in motor neurons^25^. Nevertheless, we observed phenotypes outside this system, including lipid accumulation and general disruption in fatty acid β-oxidation (Fig. 5). Interestingly, these phenotypes are observed almost a week before showing paralysis phenotype and neurodegeneration. We also demonstrated that *cpt-1* down-regulation in our FUS worm models is not limited to the neurons, while *CPT1A*, the liver variant, is also downregulated in symptomatic SOD1^G93A^ (Fig. 4f, g). Ubiquitous *knock out* of *acdh-1* and *acs-20*, both implicated in fatty acid oxidation and transport, induce neurodegeneration and exacerbate ALS-related phenotypes in our FUS models (Fig. 6). Finally, HA-114 dietary supplementation targets mostly the intestine and not the nervous system directly, while having significant effect in the neurons. However, further investigation is needed to determine if HA-114’s fatty acids are neuroprotective directly through neuronal β-oxidation or through other systems. Taken together, these results do support the involvement of several systems in ALS pathogenesis.

The inaccessibility of the CNS when patients are still alive complexify the study of metabolic changes through the disease, therefore, studying more accessible organs or systems might be the key for the discovery of biomarkers. Thus, the necessity of studying models in vivo, before and after the onset of the disease. Fundamental biology studies on the early events affecting systemic organs, like the liver, and their associated disrupted metabolism pathways should be a focus for investigation. Our investigation of bacterial strain-specific neuroprotection provides insights into neurodegeneration by identifying impaired lipid homeostasis as a feature of the disease, while also identifying potential therapeutic strategies. Importantly, severe side effects are a major reason for discontinuation of clinical drug development. Thus, probiotics may be an alternative or complementary approach for neurodegenerative diseases since chronic treatment with probiotics is associated with a low risk of side effects.

## Methods

### *C. elegans* maintenance and strains

*C. elegans* were maintained as previously described^144^. Briefly, worms were kept on NGM agar plates that were streaked with *E*. *coli* OP50, *L. rhamnosus* HA-114 or other probiotics as food source (Table 1). All the probiotics strains were kindly provided by Lallemand Health Solutions (Montreal, Canada). All assays were performed at 20 °C. The N2 Bristol strain, as well as EG1285 (oxls12 [unc-47p::GFP+lin-15(+)]), VS24 (kat-1(tm1037)), VC1011 (acdh-1(ok1489)), VC425 (elo-6(gk233)), VL749 (wwIs24[Pacdh-1::GFP; unc-119(+)]), RB754 (aak-2(ok524)), CF1038 (daf-16(mu86)), VC199 (sir-2.1(ok434)), PS3551 (hsf-1(sy441)), SJ4197 (zcIs39[dve-1p::dve-1::GFP]), IG274 (frIs7[nlp-29p::GFP,col-12p::DsRed]), SJ4100 (zcls13[hsp-6::GFP]), SJ4005 (zcIs4[hsp-4::GFP; lin-15(n765)), SJ4058 (zcls9 [hsp-60::GFP+lin-15(+)]), TJ375 (gpls1[hsp-16.2p::GFP]), AM44 (rmIs190 [F25B3.3p::Q67::CFP]), AM101 (rmIs110 [F25B3.3p::Q40::YFP]) and AM141 (rmIs133 [unc-54p::Q40::YFP]) were obtained from the *C. elegans* Genetics Center (University of Minnesota, Minneapolis), which is funded by NIH Office of Research Infrastructure Programs (P40 OD010440). VC425, VC1011 and VC199 was provided by the *C. elegans* Reverse Genetics Core Facility at the University of British Colombia, which is part of the international *C. elegans* Gene Knockout Consortium^145^. RB754 was provided by the *C. elegans* Gene Knockout Project at the Oklahoma Medical Research Foundation, which is also part of the international *C. elegans* Gene Knockout Consortium^145^. FX03278 (*acs-20(tm3278)*) was obtained from S. Mitani and the Japanese National BioResource Project (Tokyo, Japan). PHX1928 (*acdh-10(syb1928)*) was made by SunyBiotech Co., Ltd by introducing a nonsense mutation in the gene. Mutant strains were outcrossed to N2 4 times before use. Other *C. elegans* strains were obtained by crossing. Homozygosity of all genotypes was confirmed by PCR or sequencing. Oligo sequences used for genotyping can be found in Supplementary Table 5.

Transgenic lines expressing mutant TDP-43^A315T^, wild-type TDP-43 (TDP-43^WT^), mutant FUS^S57Δ^ and wild-type FUS (FUS^WT^) were created as previously described^25^. Several strains showing comparable phenotypes and transgene expression levels were kept and the strains used in this study include: XQ98 (*xqIs98 [unc47p::FUS*^*S57Δ*^*;unc-119(+)]*), XQ173 (*xqIs173 [unc-47p::FUS*^*WT*^*; unc-119(+)]*), XQ132 (*xqIs132* [*unc-47p::TDP-43*^*WT*^*; unc-119(+)]*) and XQ133 (*xqIs133 [unc-47p::TDP-43*^*A315T*^*; unc-119(+)]*).

### Paralysis assay on solid media

Briefly, 40 age-synchronized L4 worms were transferred to NGM plates and scored daily for paralysis, from day 1 to day 12 of adulthood. Animals were counted as paralyzed if they failed to move upon prodding with a worm pick. Worms were scored as dead if they failed to move their head after being prodded on the nose and showed no pharyngeal pumping. All experiments were conducted at 20 °C and in triplicates, three times. Some experiments were conducted by dissolving Etomoxir (40 μM, Medchem express) or Paraquat (250 μM, Sigma-Aldrich) into the NGM plates.

### Lifespan assay

Approximately 40 age-synchronized L4 worms were transferred NGM plates streaked with OP50 or HA-114 and tested every 2 days from day 1 adult until death. Worms were scored as dead if they failed to respond to tactile stimulus and showed no spontaneous movement or response when prodded. Dead worms displaying internally hatched progeny or extruded gonads or worms that crawled off the plate were excluded. All experiments were conducted at 20 °C and in triplicates. Some experiments were conducted by dissolving Etomoxir (Medchem express) into the NGM plates at a concentration of 40 μM.

### Neurodegeneration assay

For scoring of neuronal processes for gaps or breakage, worms were selected at day 9 of adulthood for visualization of motor neuron in vivo. Animals were immobilized in 5 mM levamisole dissolved in M9 and mounted on slides with 2% agarose pads. GFP was visualized at 505 nm using a Zeiss Axio Imager M2 microscope, using a ×20 objective and a 1.5 Optovar. The software used was AxioVs40 4.8.2.0. At least one hundred worms were scored per condition, over 4 distinct experiments.

### Aggregation assay

Briefly, worms were synchronized and fed with OP50 or *Lacticaseibacillus rhamnosus* HA-114 until they reach L4 stage or day 1 adult. Visible aggregates were scored in each worm and animals were divided into three categories of aggregation: light (<5), moderate (between 5 and 15) and strong (>15). Between 215 and 240 worms per condition were tested over 3 trials.

### *C. elegans* fluorescence microscopy

For visualization of GFP worms, a 5 mM solution of levamisole diluted in M9 was used for immobilization. Animals were mounted on slides with 2% agarose pads. Fluorescent expression for quantification was visualized with a Zeiss microscope AxioObserver Z1. The software used was AxioVs40 4.8.2.0. Thirty day-1 adult worms were visualized per condition, over three different experiments. Image processing and quantification were done with Fiji. To compare fluorescence, we calculated the changes in the ratio (size/intensity of fluorescence).

### Oil Red O staining, imaging and quantification

Oil red O staining was conducted by as previously reported, but omitting the freeze-thaw steps and the MRWB-PFA permeabilization^146,147^. Briefly, Oil Red O stock solution was made at a concentration of 10 mM and balanced over 2 days. Working solution was freshly made before each use at a concentration of 6 mM and filtered. Age-synchronized day 1 adults worms were dehydrated in PBS/isopropanol 60%/0.01% Triton-X for 15 min at RT and then stained overnight at RT with Oil red O working solution. Worms were washed 3 times with PBS/0,01% Triton-X and mounted on slides with 2% agarose pads. Oil red O staining was visualized using an Olympus brightfield microscope with a DP21 microscope digital system and a ×10 objective. Images were quantified using the Fiji (ImageJ) software. Integrated density was used as the primary measure and relative percentage of signal was calculated. ORO stain was quantified in 55–153 animals per condition, over 3 different sets of experiments.

### Gene expression and RNA-Seq analysis

Total RNA was obtained from synchronized adult day 1 *C. elegans* and liver from symptomatic (P120) mice using the “RNA extraction for RNA-Seq” protocol from the Bowdish Lab (McMaster University, Hamilton, Canada), quantified photometrically with a NanoPhotometer (Implen) and stored at −80 °C until further use. For gene expression analysis, cDNA from either 800 ng (cpt-1 qPCR experiment) or 200 ng (acdh-1 qPCR experiment) total RNA was generated using the Superscript Vilo cDNA synthesis kit (Thermo Fischer Scientific). Samples were used, undiluted, and yielded a CT value between 15 and 28. Gene expression was analyzed using TaqMan Gene Expression Assays (Applied Biosystems) and a QuantStudio 3 Real-Time PCR System (Thermo Fisher). Data were normalized to the housekeeping gene *ama-1* (for *C.elegans*) or *Polr2a* (for mice) and analyzed using the Δ/Δ-CT method. All experiments were made at least in triplicates, three times.

For RNA-Seq, library preparation and sequencing was made at the Institute for Research in Immunology and Cancer (IRIC) Genomics Platform and analyzed by the bioinformatics service (Université de Montréal). 500 ng of total RNA was used for library preparation. Quality of total RNA was assessed with the BioAnalyzer Nano (Agilent) and all samples had a RIN above 7. Library preparation was done with the KAPA mRNAseq hyperprep standed kit (KAPA, Cat no. KK8421). Ligation was made with 1.4 nM final concentration of Illumina Truseq index and 16 PCR cycles was required to amplify cDNA libraries. Libraries were quantified by QuBit and average library length was evaluated with the BioAnalyzer DNA1000. All libraries were diluted to 10 nM and normalized by qPCR using the KAPA library quantification kit (KAPA; Cat no. KK4973). Libraries were pooled to equimolar concentration. Sequencing will be performed with the Illumina Nextseq500 using the Nextseq High Output 75 cycles (1 × 75 bp) using 2 pM of the pooled library. Around 25 M single-end reads was generated per sample. Sequences were trimmed for sequencing adapters and low quality 3’ bases using Trimmomatic version 0.35^148^ and aligned to the reference *C.elegans* genome version WBcel235 (gene annotation from Ensembl version 84) using STAR version 2.5.1b^149^. Gene expressions were obtained both as readcount directly from STAR as well as computed using RSEM^150^ in order to obtain gene and transcript level expression, either in TPM or FPKM values. DESeq2 version 1.6.2^151^ was then used to identify differentially expressed genes. Sample clustering based on normalized log read counts produces the following hierarchy of samples. To identify significant biological pathways in which differentially expressed genes were enriched, gene-ontology (GO) term analysis was conducted using PANTHER 11^152–154^. RNA-Seq raw data are publicly available (GEO accession: GSE189988; SRA study: SRP348888).

### Bioinformatics analysis

The RNA-sequencing data from human frontal cortex and cerebellum from Prudencio et *al*. dataset^60^ (GEO accession: GSM1642314; SRA study:SRP056477) a were aligned using HiSAT2 against reference genome Hg38. Read counts were obtained HTSeq-count and differential expression analysis were performed with the Bioconductor R package DESeq2.

### Fatty acids extraction and measurements

A fatty acid extraction kit (MAK174, Sigma) was used to perform the extraction. Pellets of OP50 and *L. rhamnosus* HA-114 were homogenized in the provided extraction buffer and vortexed. The extraction was done as described in the provided protocol. All fatty acids extractions were freshly made on the day of their use. Samples were quantified using colorimetric assay as described in the provided protocol of the Free fatty acids quantification kit used (MAK044-1KT, Sigma). Experiments were conducted by adding OP50 or *L. rhamnosus* HA-114 fatty acids extracts in OP50 at a concentration of 400 nM before streaking the plates.

### Protein extraction and measurements

OP50 and L. rhamnosus HA-114 pellets were lysed in RIPA buffer (150 mM NaCl, 50 mM Tris pH 7.4, 1% Triton X-100, 0.1% SDS and 1% sodium deoxycholate) containing 0.1% protease inhibitors (10 mg/ml leupeptin, 10 mg/ml pepstatin A and 10 mg/ml chymostatin LPC; 1/1000).Pellets were passed through a 27.5-gauge syringe ten times, sonicated for 5 min, and centrifuged at 16,000 × *g* for 10 min at 4 °C. Supernatants were collected in 1.5 ml tubes. The supernatants were quantified using the BCA protein assay kit (Thermo Scientific) following the manufacturer’s protocol and instructions. Samples were stored at −80 °C until their use. Experiments were conducted by adding 500 μg/ml of OP50 or *L. rhamnosus* HA-114 proteins extracts on OP50-streaked plates.

### Lipidomics experiments and analysis

Lipid extraction, liquid chromatography-mass spectrometry (LC-MS) analysis and data processing were done as previously described^155^. In brief, lipids were extracted from 6 replicates of three different bacteria strain (OP50, HA-114, R0011) and spiked with six internal standards: LPC 13:0, PC19:0/19:0, PC14:0/14:0, PS12:0/12:0, PG15:0/15:0, and PE17:0/17:0 (Avanti Polar Lipids Inc, Alabaster, USA).

Samples were injected into a 1290 Infinity High-pressure liquid chromatography (HPLC) coupled with a 6530 Accurate Mass Quadrupole Time-of-Flight (Q-TOF) (Agilent Technologies Inc., Santa Clara, USA) via a dual electrospray ionization (ESI) source in positive and negative ionization. Volumes of injection were adjusted for similar total ion chromatogram (TIC), giving an equivalent number of bacteria injected of: 3.00E09 +/− 4.61E06, 1.74E09 +/− 2.92E06 and 2.07E09 +/− 3.76E06 for OP50, HA-114 and R0011 respectively, for positive ionization. The double amount was injected for negative ionization. Elution of lipids was assessed on a Zorbax Eclipse plus column (C18, 2.1 × 100 mm, 1.8 μm, Agilent Technologies Inc.) maintained at 40 °C using a 83 min chromatographic gradient of solvent A (0.2% formic acid and 10 mM ammonium formate in water) and B (0.2% formic acid and 5 mM ammonium formate in methanol/acetonitrile/methyl tert-butyl ether [MTBE], 55:35:10 [v/v/v]).

A list of MS features, characterized by mass and retention time, was extracted using Mass Hunter B.06.00 (Agilent Technologies Inc.). Subsequent data mining was achieved using an in-house script that applies alignment of the chromatographic runs, following by the different next steps. First, the data set was divided in three data sets for which each one contains only 2 strains. For each dataset, features with 100 % of missing values for one strain were first removed. Then only features with 80% of presence in one of the two strains were kept. Imputation of missing value by 90% of lowest value for each feature and normalization with cyclic loess on scale data were then applied. Statistical analysis of each feature was achieved using regression analysis in R, with Storey correction for multiple comparisons using the *Q*-value package from Bioconductor. A significant threshold was set at *p* value = 0.01, corresponding to a *Q*-value <0.05 for comparison HA-114 vs. R0011, the two most similar strains and associated with a fold change>1.5 or <0.667 between the two groups.

For lipid annotations, the open database Metlin was used in a first step, to annotate features based on their accurate mass (<5 ppm) to the following subclasses: free fatty acids (FFA), diacylglycerophosphoethanolamines (PE), diacylglycerophosphoglycerols (PG), diacylglycerophosphocholines (PC), Monoglycosyldiacylglycerols (MGDG), diglycosyldiacylglycerols (DGDG), Lysl-diacylglycerophosphoglycerols (LPG). A second step of confirmation was used by the presence of MS signal for a same retention time (<30 s) between positive and negative ionization, considering the following mass to charge (*m*/*z*): (i) FFA: [M + NH4]+ and [M-H]^−^, (ii) PE: [M + H]^+^ and [M-H]^−^, (iii) PG, [M + NH4]^+^ and [M-H]^−^, (iv) PC: [M + H]^+^ and [M + FA-H]^−^, (v) MGDG and DGDG: [M + NH4]^+^, (vi) LPG: [M-H]. All these annotations were not confirmed by MS/MS or standards. Raw data and untargeted lipidomic results analysis are available in Supplementary Data files 1 and 2.

### Statistics and reproducibility

All experiments were repeated at least three times. Quantitative data were expressed as mean ± SEM. GraphPad Prism v8 software was used for all statistical analyses, except the bioinformatics analysis, where R was used. All statistic tests and experimental *n* are clearly indicated in figure legends.

### Reporting summary

Further information on research design is available in the Nature Portfolio Reporting Summary linked to this article.

## Supplementary information

Peer Review File

Supplementary Information

Description of Additional Supplementary Files

Supplementary Data 1

Supplementary Data 2

Supplementary Data 3

Reporting Summary

## Data Availability

The authors declare that all relevant data supporting the findings of this study are available within the paper and its supplementary information files. Lipidomics data sets (Fig. 7 and S16) are available in Supplementary Data files 1 and 2. RNA-Seq data generated for this manuscript (Figs. 3 and S6) have been deposited on NCBI’s Gene Expression Omnibus (GEO) (GSE189988; SRA study SRP348888; https://www.ncbi.nlm.nih.gov/geo/query/acc.cgi?acc=GSE189988). RNA-Seq data used for this manuscript (Fig. 4 and S14) are publicly available on NCBI’s Gene Expression Omnibus (GEO) (GSM1642314; SRA study: SRP056477; https://www.ncbi.nlm.nih.gov/geo/query/acc.cgi?acc=GSE67196)^60^. Source data for all figures are provided with the paper (Figs. 1–8 and S1–S16) in Supplementary Data file 3. Any remaining raw data will be available from the corresponding author upon reasonable request.

## References

[1] Lynch SV, Pedersen O (2016). The human intestinal microbiome in health and disease. N. Engl. J. Med..

[2] Astafurov K (2014). Oral microbiome link to neurodegeneration in glaucoma. PLoS ONE.

[3] Clark RI, Walker DW (2017). Role of gut microbiota in aging-related health decline: insights from invertebrate models. Cell. Mol. Life Sci..

[4] Wu S, Yi J, Zhang Y-G, Zhou J, Sun J (2015). Leaky intestine and impaired microbiome in an amyotrophic lateral sclerosis mouse model. Physiol. Rep..

[5] Dopkins N, Nagarkatti PS, Nagarkatti M (2018). The role of gut microbiome and associated metabolome in the regulation of neuroinflammation in multiple sclerosis and its implications in attenuating chronic inflammation in other inflammatory and autoimmune disorders. Immunology.

[6] Vogt NM (2017). Gut microbiome alterations in Alzheimer’s disease. Sci. Rep..

[7] Mulak A, Bonaz B (2015). Brain-gut-microbiota axis in Parkinson’s disease. World J. Gastroenterol..

[8] Sherwin E, Dinan TG, Cryan JF (2018). Recent developments in understanding the role of the gut microbiota in brain health and disease. Ann. NY Acad. Sci..

[9] Roy Sarkar S, Banerjee S (2019). Gut microbiota in neurodegenerative disorders. J. Neuroimmunol..

[10] Akbari E (2016). Effect of probiotic supplementation on cognitive function and metabolic status in Alzheimer’s disease: a randomized, double-blind and controlled trial. Front. Aging Neurosci..

[11] Harding A, Gonder U, Robinson SJ, Crean S, Singhrao SK (2017). Exploring the association between Alzheimer’s disease, oral health, microbial endocrinology and nutrition. Front. Aging Neurosci..

[12] Blacher E (2019). Potential roles of gut microbiome and metabolites in modulating ALS in mice. Nature.

[13] Burberry A (2020). C9orf72 suppresses systemic and neural inflammation induced by gut bacteria. Nature.

[14] Zhang Y-G (2017). Target intestinal microbiota to alleviate disease progression in amyotrophic lateral sclerosis. Clin. Ther..

[15] MacNeil LT, Watson E, Arda HE, Zhu LJ, Walhout AJM (2013). Diet-induced developmental acceleration independent of TOR and insulin in *C. elegans*. Cell.

[16] Schulenburg H, Félix M-A (2017). The natural biotic environment of *Caenorhabditis elegans*. Genetics.

[17] Watson E, MacNeil LT, Arda HE, Zhu LJ, Walhout AJM (2013). Integration of metabolic and gene regulatory networks modulates the *C. elegans* dietary response. Cell.

[18] Watson E (2014). Interspecies systems biology uncovers metabolites affecting *C. elegans* gene expression and life history traits. Cell.

[19] Gerbaba TK, Green-Harrison L, Buret AG (2018). Modeling host-microbiome interactions in *Caenorhabditis elegans*. J. Nematol..

[20] Shapira M (2017). Host–microbiota interactions in *Caenorhabditis elegans* and their significance. Curr. Opin. Microbiol..

[21] Therrien M, Parker JA (2014). Worming forward: amyotrophic lateral sclerosis toxicity mechanisms and genetic interactions in *Caenorhabditis elegans*. Front. Genet..

[22] Vérièpe J, Fossouo L, Parker JA (2015). Neurodegeneration in *C. elegans* models of ALS requires TIR-1/Sarm1 immune pathway activation in neurons. Nat. Commun..

[23] Schmeisser K, Parker JA (2018). Nicotinamide-N-methyltransferase controls behavior, neurodegeneration and lifespan by regulating neuronal autophagy. PLoS Genet..

[24] McIntire SL, Reimer RJ, Schuske K, Edwards RH, Jorgensen EM (1997). Identification and characterization of the vesicular GABA transporter. Nature.

[25] Vaccaro A (2012). Mutant TDP-43 and FUS cause age-dependent paralysis and neurodegeneration in *C. elegans*. PLoS ONE.

[26] Yoneda T (2004). Compartment-specific perturbation of protein handling activates genes encoding mitochondrial chaperones. J. Cell. Sci..

[27] Brignull HR, Moore FE, Tang SJ, Morimoto RI (2006). Polyglutamine proteins at the pathogenic threshold display neuron-specific aggregation in a pan-neuronal *Caenorhabditis elegans* model. J. Neurosci..

[28] Gidalevitz T, Ben-Zvi A, Ho KH, Brignull HR, Morimoto RI (2006). Progressive disruption of cellular protein folding in models of polyglutamine diseases. Science.

[29] Lin K, Dorman JB, Rodan A, Kenyon C (1997). daf-16: An HNF-3/forkhead family member that can function to double the life-span of *Caenorhabditis elegans*. Science.

[30] Hsu A-L, Murphy CT, Kenyon C (2003). Regulation of aging and age-related disease by DAF-16 and heat-shock factor. Science.

[31] Tissenbaum HA, Guarente L (2001). Increased dosage of a sir-2 gene extends lifespan in *Caenorhabditis elegans*. Nature.

[32] Apfeld J, O’Connor G, McDonagh T, DiStefano PS, Curtis R (2004). The AMP-activated protein kinase AAK-2 links energy levels and insulin-like signals to lifespan in *C. elegans*. Genes Dev..

[33] Morley JF, Brignull HR, Weyers JJ, Morimoto RI (2002). The threshold for polyglutamine-expansion protein aggregation and cellular toxicity is dynamic and influenced by aging in *Caenorhabditis elegans*. Proc. Natl Acad. Sci. USA.

[34] Pujol N (2008). Distinct innate immune responses to infection and wounding in the *C. elegans* epidermis. Curr. Biol..

[35] Haynes CM, Petrova K, Benedetti C, Yang Y, Ron D (2007). ClpP mediates activation of a mitochondrial unfolded protein response in *C. elegans*. Dev. Cell.

[36] Calfon M (2002). IRE1 couples endoplasmic reticulum load to secretory capacity by processing the XBP-1 mRNA. Nature.

[37] Link CD, Cypser JR, Johnson CJ, Johnson TE (1999). Direct observation of stress response in *Caenorhabditis elegans* using a reporter transgene. Cell Stress Chaperones.

[38] Rea SL, Wu D, Cypser JR, Vaupel JW, Johnson TE (2005). A stress-sensitive reporter predicts longevity in isogenic populations of *Caenorhabditis elegans*. Nat. Genet..

[39] Matey-Hernandez ML (2018). Genetic and microbiome influence on lipid metabolism and dyslipidemia. Physiol. Genomics.

[40] Schoeler M, Caesar R (2019). Dietary lipids, gut microbiota and lipid metabolism. Rev. Endocr. Metab. Disord..

[41] Dupuis L (2008). Dyslipidemia is a protective factor in amyotrophic lateral sclerosis. Neurology.

[42] Shamim A, Mahmood T, Ahsan F, Kumar A, Bagga P (2018). Lipids: an insight into the neurodegenerative disorders. Clin. Nutr. Exp..

[43] Watts JL, Ristow M (2017). Lipid and carbohydrate metabolism in *Caenorhabditis elegans*. Genetics.

[44] Mak HY, Nelson LS, Basson M, Johnson CD, Ruvkun G (2006). Polygenic control of *Caenorhabditis elegans* fat storage. Nat. Genet..

[45] Kniazeva M, Crawford QT, Seiber M, Wang C-Y, Han M (2004). Monomethyl branched-chain fatty acids play an essential role in *Caenorhabditis elegans* development. PLoS Biol..

[46] Sang L-X (2014). Heat-killed VSL#3 ameliorates dextran sulfate sodium (DSS)-induced acute experimental colitis in rats. Int. J. Mol. Sci..

[47] Sugahara H, Yao R, Odamaki T, Xiao JZ (2017). Differences between live and heat-killed bifidobacteria in the regulation of immune function and the intestinal environment. Benef. Microbes.

[48] Bulcha JT (2019). A persistence detector for metabolic network rewiring in an animal. Cell Rep..

[49] LeBlanc JG (2017). Beneficial effects on host energy metabolism of short-chain fatty acids and vitamins produced by commensal and probiotic bacteria. Microb. Cell Fact..

[50] Giese GE (2020). *Caenorhabditis elegans* methionine/S-adenosylmethionine cycle activity is sensed and adjusted by a nuclear hormone receptor. Elife.

[51] Raud B (2018). Etomoxir actions on regulatory and memory T cells are independent of Cpt1a-mediated fatty acid oxidation. Cell Metab..

[52] Yao C-H (2018). Identifying off-target effects of etomoxir reveals that carnitine palmitoyltransferase I is essential for cancer cell proliferation independent of β-oxidation. PLoS Biol..

[53] Wang Z (2015). The nuclear receptor DAF-12 regulates nutrient metabolism and reproductive growth in nematodes. PLoS Genet..

[54] Weir HJ (2017). Dietary restriction and AMPK increase lifespan via mitochondrial network and peroxisome remodeling. Cell Metab..

[55] Kim H-E (2016). Lipid biosynthesis coordinates a mitochondrial-to-cytosolic stress response. Cell.

[56] Glatz JFC, Luiken JJFP (2017). From fat to FAT (CD36/SR-B2): understanding the regulation of cellular fatty acid uptake. Biochimie.

[57] Campbell SE (2004). A novel function for fatty acid translocase (FAT)/CD36: involvement in long chain fatty acid transfer into the mitochondria. J. Biol. Chem..

[58] Anderson CM, Stahl A (2013). SLC27 fatty acid transport proteins. Mol. Asp. Med..

[59] Gurney ME (1994). Motor neuron degeneration in mice that express a human Cu,Zn superoxide dismutase mutation. Science.

[60] Prudencio M (2015). Distinct brain transcriptome profiles in C9orf72-associated and sporadic ALS. Nat. Neurosci..

[61] Wong MW (2017). Dysregulation of lipids in Alzheimer’s disease and their role as potential biomarkers. Alzheimers Dement..

[62] Abdel-Khalik J (2017). Defective cholesterol metabolism in amyotrophic lateral sclerosis. J. Lipid Res..

[63] Fromenty B, Robin MA, Igoudjil A, Mansouri A, Pessayre D (2004). The ins and outs of mitochondrial dysfunction in NASH. Diabetes Metab..

[64] Kage-Nakadai E (2010). Two very long chain fatty acid acyl-CoA synthetase genes, acs-20 and acs-22, have roles in the cuticle surface barrier in *Caenorhabditis elegans*. PLoS ONE.

[65] Song J (2019). Neuroprotective effects of oleic acid in rodent models of cerebral ischaemia. Sci. Rep..

[66] Lee AY, Lee MH, Lee S, Cho EJ (2018). Neuroprotective effect of alpha-linolenic acid against Aβ-mediated inflammatory responses in C6 glial cell. J. Agric. Food Chem..

[67] Xie C, Prasad AA (2020). Probiotics treatment improves hippocampal dependent cognition in a rodent model of Parkinson’s disease. Microorganisms.

[68] Vishwanath VA (2016). Fatty acid beta-oxidation disorders: a brief review. Ann. Neurosci..

[69] Lee S-J, Zhang J, Choi AMK, Kim HP (2013). Mitochondrial dysfunction induces formation of lipid droplets as a generalized response to stress. Oxid. Med. Cell Longev..

[70] Cogliati S (2020). *Bacillus subtilis* delays neurodegeneration and behavioral impairment in the Alzheimer’s disease model *Caenorhabditis elegans*. J. Alzheimers Dis..

[71] Goya ME (2020). Probiotic *Bacillus subtilis* protects against α-synuclein aggregation in *C. elegans*. Cell Rep..

[72] Urrutia A (2020). Bacterially produced metabolites protect *C. elegans* neurons from degeneration. PLoS Biol..

[73] Grompone G (2012). Anti-inflammatory *Lactobacillus rhamnosus* CNCM I-3690 strain protects against oxidative stress and increases lifespan in *Caenorhabditis elegans*. PLoS ONE.

[74] Vercruysse P, Vieau D, Blum D, Petersén Å, Dupuis L (2018). Hypothalamic alterations in neurodegenerative diseases and their relation to abnormal energy metabolism. Front. Mol. Neurosci..

[75] Ahmed RM (2016). Amyotrophic lateral sclerosis and frontotemporal dementia: distinct and overlapping changes in eating behaviour and metabolism. Lancet Neurol..

[76] Jawaid A, Khan R, Polymenidou M, Schulz PE (2018). Disease-modifying effects of metabolic perturbations in ALS/FTLD. Mol. Neurodegener..

[77] Nodera H (2015). Frequent hepatic steatosis in amyotrophic lateral sclerosis: Implication for systemic involvement. Neurol. Clin. Neurosci..

[78] Dodge JC (2020). Neutral lipid cacostasis contributes to disease pathogenesis in amyotrophic lateral sclerosis. J. Neurosci..

[79] Lee SH, Yang EJ (2018). Relationship between liver pathology and disease progression in a murine model of amyotrophic lateral sclerosis. Neurodegener. Dis..

[80] Kim D-G (2016). Non-alcoholic fatty liver disease induces signs of Alzheimer’s disease (AD) in wild-type mice and accelerates pathological signs of AD in an AD model. J. Neuroinflammation.

[81] Gupta G, Qin H, Song J (2012). Intrinsically unstructured domain 3 of hepatitis C virus NS5A forms a ‘fuzzy complex’ with VAPB-MSP domain which carries ALS-causing mutations. PLoS ONE.

[82] Zolkipli Z (2012). Abnormal fatty acid metabolism in spinal muscular atrophy may predispose to perioperative risks. Eur. J. Paediatr. Neurol..

[83] Nash LA, Burns JK, Chardon JW, Kothary R, Parks RJ (2016). Spinal muscular atrophy: more than a disease of motor neurons?. Curr. Mol. Med..

[84] Goutman SA (2020). Untargeted metabolomics yields insight into ALS disease mechanisms. J. Neurol. Neurosurg. Psychiatr..

[85] Mohassel P (2021). Childhood amyotrophic lateral sclerosis caused by excess sphingolipid synthesis. Nat. Med..

[86] Jang HJ (2021). Matrix-assisted laser desorption/ionization mass spectrometry imaging of phospholipid changes in a Drosophila model of early amyotrophic lateral sclerosis. J. Am. Soc. Mass Spectrom..

[87] Fernández-Beltrán LC (2021). A transcriptomic meta-analysis shows lipid metabolism dysregulation as an early pathological mechanism in the spinal cord of SOD1 mice. Int. J. Mol. Sci..

[88] Burg T, Rossaert E, Moisse M, Van Damme P, Van Den Bosch L (2021). Histone deacetylase inhibition regulates lipid homeostasis in a mouse model of amyotrophic lateral sclerosis. Int. J. Mol. Sci..

[89] Stella R (2021). Perturbations of the proteome and of secreted metabolites in primary astrocytes from the hSOD1(G93A) ALS mouse model. Int. J. Mol. Sci..

[90] Rossaert E (2019). Restoration of histone acetylation ameliorates disease and metabolic abnormalities in a FUS mouse model. Acta Neuropathol. Commun..

[91] Lawton KA (2014). Plasma metabolomic biomarker panel to distinguish patients with amyotrophic lateral sclerosis from disease mimics. Amyotroph. Lateral Scler. Frontotemporal Degener..

[92] Lawton KA (2012). Biochemical alterations associated with ALS. Amyotroph. Lateral Scler..

[93] Sarraf P (2021). The correlation of the serum level of L-carnitine with disease severity in patients with Amyotrophic lateral sclerosis. J. Clin. Neurosci..

[94] Blasco H (2017). Lipidomics reveals cerebrospinal-fluid signatures of ALS. Sci. Rep..

[95] Kira Y, Nishikawa M, Ochi A, Sato E, Inoue M (2006). L-carnitine suppresses the onset of neuromuscular degeneration and increases the life span of mice with familial amyotrophic lateral sclerosis. Brain Res..

[96] Beghi E (2013). Randomized double-blind placebo-controlled trial of acetyl-L-carnitine for ALS. Amyotroph. Lateral Scler. Frontotemporal Degener..

[97] Manzo E (2018). Medium-chain fatty acids, beta-hydroxybutyric acid and genetic modulation of the carnitine shuttle are protective in a drosophila model of ALS based on TDP-43. Front. Mol. Neurosci..

[98] Vande Velde C, Miller TM, Cashman NR, Cleveland DW (2008). Selective association of misfolded ALS-linked mutant SOD1 with the cytoplasmic face of mitochondria. Proc. Natl Acad. Sci. USA.

[99] Pasinelli P (2004). Amyotrophic lateral sclerosis-associated SOD1 mutant proteins bind and aggregate with Bcl-2 in spinal cord mitochondria. Neuron.

[100] Ruan L (2017). Cytosolic proteostasis through importing of misfolded proteins into mitochondria. Nature.

[101] Trabjerg MS (2021). Downregulating carnitine palmitoyl transferase 1 affects disease progression in the SOD1 G93A mouse model of ALS. Commun. Biol..

[102] Tefera TW (2016). Triheptanoin protects motor neurons and delays the onset of motor symptoms in a mouse model of amyotrophic lateral sclerosis. PLoS ONE.

[103] Zhao Z (2006). A ketogenic diet as a potential novel therapeutic intervention in amyotrophic lateral sclerosis. BMC Neurosci..

[104] Ari C (2014). Metabolic therapy with Deanna Protocol supplementation delays disease progression and extends survival in amyotrophic lateral sclerosis (ALS) mouse model. PLoS ONE.

[105] Zhao W (2012). Caprylic triglyceride as a novel therapeutic approach to effectively improve the performance and attenuate the symptoms due to the motor neuron loss in ALS disease. PLoS ONE.

[106] Nakamura R (2020). A multi-ethnic meta-analysis identifies novel genes, including ACSL5, associated with amyotrophic lateral sclerosis. Commun. Biol..

[107] Iacoangeli A (2020). Genome-wide meta-analysis finds the ACSL5-ZDHHC6 locus is associated with ALS and links weight loss to the disease genetics. Cell Rep..

[108] Dalakas MC, Hatazawa J, Brooks RA, Di Chiro G (1987). Lowered cerebral glucose utilization in amyotrophic lateral sclerosis. Ann. Neurol..

[109] Hatazawa J, Brooks RA, Dalakas MC, Mansi L, Di Chiro G (1988). Cortical motor-sensory hypometabolism in amyotrophic lateral sclerosis: a PET study. J. Comput. Assist. Tomogr..

[110] Ludolph AC (1992). Frontal lobe function in amyotrophic lateral sclerosis: a neuropsychologic and positron emission tomography study. Acta Neurol. Scand..

[111] Browne SE (2006). Bioenergetic abnormalities in discrete cerebral motor pathways presage spinal cord pathology in the G93A SOD1 mouse model of ALS. Neurobiol. Dis..

[112] Miyazaki K (2012). Early and progressive impairment of spinal blood flow-glucose metabolism coupling in motor neuron degeneration of ALS model mice. J. Cereb. Blood Flow Metab..

[113] Reyes ET, Perurena OH, Festoff BW, Jorgensen R, Moore WV (1984). Insulin resistance in amyotrophic lateral sclerosis. J. Neurol. Sci..

[114] Pradat P-F (2010). Impaired glucose tolerance in patients with amyotrophic lateral sclerosis. Amyotroph. Lateral Scler..

[115] Mariosa D, Kamel F, Bellocco R, Ye W, Fang F (2015). Association between diabetes and amyotrophic lateral sclerosis in Sweden. Eur. J. Neurol..

[116] Jawaid A, Brown JA, Schulz PE (2015). Diabetes mellitus in amyotrophic lateral sclerosis: Dr. Jekyll or Mr. Hyde?. Eur. J. Neurol..

[117] Palamiuc L (2015). A metabolic switch toward lipid use in glycolytic muscle is an early pathologic event in a mouse model of amyotrophic lateral sclerosis. EMBO Mol. Med..

[118] Manzo E (2019). Glycolysis upregulation is neuroprotective as a compensatory mechanism in ALS. Elife.

[119] Simpson EP, Henry YK, Henkel JS, Smith RG, Appel SH (2004). Increased lipid peroxidation in sera of ALS patients: a potential biomarker of disease burden. Neurology.

[120] Fergani A (2007). Increased peripheral lipid clearance in an animal model of amyotrophic lateral sclerosis. J. Lipid Res..

[121] Kim CH, Younossi ZM (2008). Nonalcoholic fatty liver disease: a manifestation of the metabolic syndrome. Cleve Clin. J. Med..

[122] Blasco H (2010). 1H-NMR-based metabolomic profiling of CSF in early amyotrophic lateral sclerosis. PLoS ONE.

[123] Kumar A (2010). Metabolomic analysis of serum by (1) H NMR spectroscopy in amyotrophic lateral sclerosis. Clin. Chim. Acta.

[124] Dorst J (2011). Patients with elevated triglyceride and cholesterol serum levels have a prolonged survival in amyotrophic lateral sclerosis. J. Neurol..

[125] Gallo V (2013). Prediagnostic body fat and risk of death from amyotrophic lateral sclerosis: the EPIC cohort. Neurology.

[126] Schmitt F, Hussain G, Dupuis L, Loeffler J-P, Henriques A (2014). A plural role for lipids in motor neuron diseases: energy, signaling and structure. Front. Cell Neurosci..

[127] Rafiq MK, Lee E, Bradburn M, McDermott CJ, Shaw PJ (2015). Effect of lipid profile on prognosis in the patients with amyotrophic lateral sclerosis: insights from the olesoxime clinical trial. Amyotroph. Lateral Scler. Frontotemporal Degener..

[128] Izumi, Y. et al. Frequent hepatic steatosis in ALS: implication for systemic involvement (P6.098). *Neurology* **84**, P6.098 (2015).

[129] Hollinger SK, Okosun IS, Mitchell CS (2016). Antecedent disease and amyotrophic lateral sclerosis: what is protecting whom?. Front. Neurol..

[130] Afifi AK, Aleu FP, Goodgold J, MacKay B (1966). Ultrastructure of atrophic muscle in amyotrophic lateral sclerosis. Neurology.

[131] Sasaki S, Iwata M (1996). Ultrastructural study of synapses in the anterior horn neurons of patients with amyotrophic lateral sclerosis. Neurosci. Lett..

[132] Siklós L (1996). Ultrastructural evidence for altered calcium in motor nerve terminals in amyotropic lateral sclerosis. Ann. Neurol..

[133] Wiedemann FR (1998). Impairment of mitochondrial function in skeletal muscle of patients with amyotrophic lateral sclerosis. J. Neurol. Sci..

[134] Dupuis L (2003). Up-regulation of mitochondrial uncoupling protein 3 reveals an early muscular metabolic defect in amyotrophic lateral sclerosis. FASEB J..

[135] Echaniz-Laguna A (2006). Muscular mitochondrial function in amyotrophic lateral sclerosis is progressively altered as the disease develops: a temporal study in man. Exp. Neurol..

[136] Vielhaber S (2000). Mitochondrial DNA abnormalities in skeletal muscle of patients with sporadic amyotrophic lateral sclerosis. Brain.

[137] Krasnianski A (2005). Mitochondrial changes in skeletal muscle in amyotrophic lateral sclerosis and other neurogenic atrophies. Brain.

[138] Song W, Song Y, Kincaid B, Bossy B, Bossy-Wetzel E (2013). Mutant SOD1G93A triggers mitochondrial fragmentation in spinal cord motor neurons: neuroprotection by SIRT3 and PGC-1α. Neurobiol. Dis..

[139] Onesto E (2016). Gene-specific mitochondria dysfunctions in human TARDBP and C9ORF72 fibroblasts. Acta Neuropathol. Commun..

[140] Allen SP, Duffy LM, Shaw PJ, Grierson AJ (2015). Altered age-related changes in bioenergetic properties and mitochondrial morphology in fibroblasts from sporadic amyotrophic lateral sclerosis patients. Neurobiol. Aging.

[141] Konrad C (2017). Fibroblast bioenergetics to classify amyotrophic lateral sclerosis patients. Mol. Neurodegener..

[142] Joshi AU (2018). Inhibition of Drp1/Fis1 interaction slows progression of amyotrophic lateral sclerosis. EMBO Mol. Med..

[143] Walczak J (2019). Distinction of sporadic and familial forms of ALS based on mitochondrial characteristics. FASEB J..

[144] Stiernagle, T. Maintenance of *C. elegans*. *WormBook* 1–11 (2006). 10.1895/wormbook.1.101.1 PMC4781397 18050451

[145] C. elegans Deletion Mutant Consortium. (2012). large-scale screening for targeted knockouts in the *Caenorhabditis elegans* genome. G3.

[146] Soukas AA, Kane EA, Carr CE, Melo JA, Ruvkun G (2009). Rictor/TORC2 regulates fat metabolism, feeding, growth, and life span in *Caenorhabditis elegans*. Genes Dev..

[147] O’Rourke EJ, Soukas AA, Carr CE, Ruvkun G (2009). *C. elegans* major fats are stored in vesicles distinct from lysosome-related organelles. Cell Metab..

[148] Bolger AM, Lohse M, Usadel B (2014). Trimmomatic: a flexible trimmer for Illumina sequence data. Bioinformatics.

[149] Dobin A (2012). STAR: ultrafast universal RNA-seq aligner. Bioinformatics.

[150] Li B, Dewey CN (2011). RSEM: accurate transcript quantification from RNA-Seq data with or without a reference genome. BMC Bioinformatics.

[151] Love MI, Huber W, Anders S (2014). Moderated estimation of fold change and dispersion for RNA-seq data with DESeq2. Genome Biol..

[152] Ashburner M (2000). Gene ontology: tool for the unification of biology. The Gene Ontology Consortium. Nat. Genet..

[153] The Gene Ontology Consortium. (2019). The Gene Ontology Resource: 20 years and still GOing strong. Nucleic Acids Res..

[154] Mi H (2017). PANTHER version 11: expanded annotation data from Gene Ontology and Reactome pathways, and data analysis tool enhancements. Nucleic Acids Res..

[155] Forest A (2018). Comprehensive and reproducible untargeted lipidomic workflow using LC-QTOF validated for human plasma analysis. J. Proteome Res..

