# Supplementary material for: Fatty acids derived from the probiotic Lacticaseibacillus rhamnosus HA-114 suppress age-dependent neurodegeneration
Source: Commun Biol. 2022 Dec 7;5:1340. doi: 10.1038/s42003-022-04295-8 (PMC9729297; doi:10.1038/s42003-022-04295-8)
Supplement: Supplementary file 2 — Supplementary Information [file 42003_2022_4295_MOESM2_ESM.pdf]

Supplementary information for

Fatty acids derived from the probiotic *Lacticaseibacillus rhamnosus* HA-114 suppress age-dependent neurodegeneration

Authors: Audrey Labarre[1, 2], Ericka Guitard[1, 2], Gilles Tossing[1, 2], Anik Forest[3], Eric Bareke[2], Marjorie Labrecque[2,4], Martine Tétreault[1,2], Matthieu Ruiz[3,5] & J Alex Parker[1, 2 *]

Affiliations:

[1] Département de neurosciences, Université de Montréal, Montréal, Canada

[2] Centre de recherche du centre hospitalier de l'Université de Montréal (CRCHUM), Montréal, Canada.

[3] Centre de recherche, Institut de Cardiologie de Montréal, Montréal, Québec, Canada.

[4] Département de biochimie, Université de Montréal, Montréal, Canada

[5] Département de nutrition, Université de Montréal, Montréal, Canada

*Correspondence to: ja.parker@umontreal.ca

Contents

Supplementary figure 1 Probiotics screen identified *Lacticaseibacillus rhamnosus* HA-114 as a neuroprotective bacterial strain.

Supplementary figure 2. *Lacticaseibacillus rhamnosus* HA-114 is the only neuroprotective *rhamnosus* strain but does not extend lifespan

Supplementary figure 3. *Lacticaseibacillus rhamnosus* HA-114 prevents damage from chronic oxidative stress.

Supplementary figure 4. HA-114 does not require classic stress and metabolic pathways in FUS$^{S57\Delta}$ worms for neuroprotection.

Supplementary figure 5. HA-114 does not activate classic stress pathways in *C. elegans*

Supplementary figure 6. *Bifidobacterium animalis subsp. lactis* B94 induces differential genes expression in *C. elegans*.

Supplementary figure 7. HA-114 modulate *acdh-1* expression through aging in mutant FUS worms.

Supplementary figure 8. *acdh-10* is not required for neuroprotection provided by HA-114 in FUS$^{S57\Delta}$ worms.

Supplementary figure 9. *kat-1* and *elo-6* are essential for neuroprotection provided by HA-114 in TDP-43^{A315T} ALS model.

Supplementary figure 10. Simplified pathway of fatty acids metabolism and β-oxidation in *C. elegans* and *H. sapiens*.

Supplementary figure 11. *acdh-1* is activated by HA-114' fatty acids.

Supplementary figure 12. Modulating *acdh-1* pathway partially affects HA-114 neuroprotective effect.

Supplementary figure 13. Etomoxir does not cause oxidative stress in *C. elegans*.

Supplementary figure 13. Etomoxir does not cause oxidative stress in *C. elegans*.

Supplementary figure 14: Genes associated with β-oxidation are differentially expressed in the cerebellum and the frontal cortex of c9ALS patients.

Supplementary figure 15. Fatty acids from HA-114 modulate lipid accumulation in worm age-dependent neurodegeneration models.

Supplementary figure 16. Free fatty acids (FFA) annotated from OP50, HA-114 and R0011 bacterial strains.

Supplementary Table 1. List of upregulated genes in N2 worms fed with HA-114 (compared to OP50)

Supplementary Table 2: List of downregulated genes in N2 worms fed with HA-114 (compared to OP50)

Supplementary Table 3: List of upregulated genes in N2 worms fed with B94 (compared to OP50)

Supplementary Table 4: List of downregulated genes in N2 worms fed with B94 (compared to OP50)

Supplementary Table 5: List of oligo sequences used for genotyping

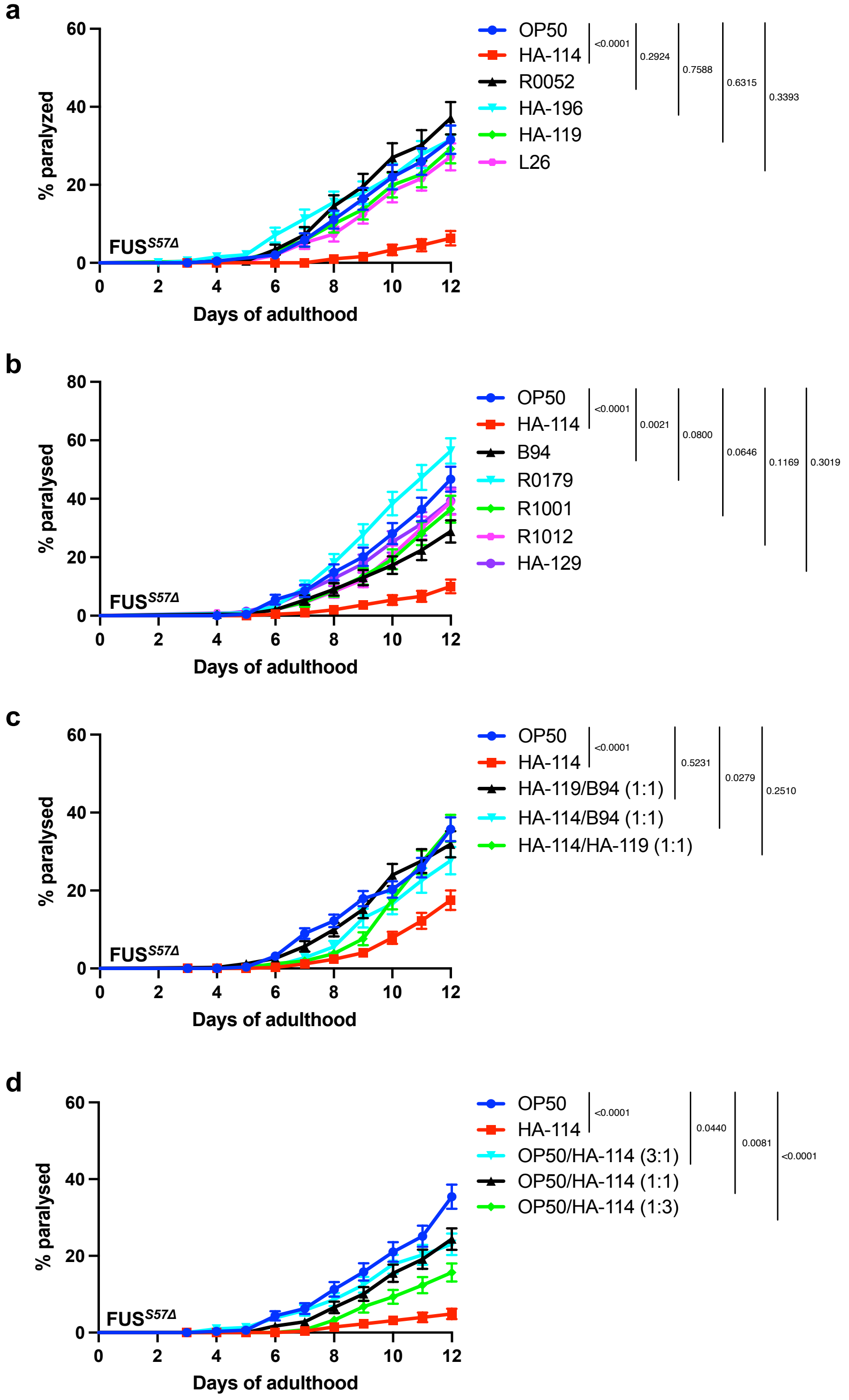

a
60
40
20
0
% paralyzed
FUS$^{S57\Delta}$
0 2 4 6 8 10 12
Days of adulthood
OP50
HA-114
R0052
HA-196
HA-119
L26
<0.0001
0.2924
0.7588
0.6315
0.3393
b
80
60
40
20
0
% paralysed
FUS$^{S57\Delta}$
0 2 4 6 8 10 12
Days of adulthood
OP50
HA-114
B94
R0179
R1001
R1012
HA-129
<0.0001
0.0021
0.0800
0.0646
0.1169
0.3019
c
60
40
20
0
% paralysed
FUS$^{S57\Delta}$
0 2 4 6 8 10 12
Days of adulthood
OP50
HA-114
HA-119/B94 (1:1)
HA-114/B94 (1:1)
HA-114/HA-119 (1:1)
<0.0001
0.5231
0.0279
0.2510
d
60
40
20
0
% paralysed
FUS$^{S57\Delta}$
0 2 4 6 8 10 12
Days of adulthood
OP50
HA-114
OP50/HA-114 (3:1)
OP50/HA-114 (1:1)
OP50/HA-114 (1:3)
<0.0001
0.0440
0.0081
<0.0001

Supplementary figure 1. Probiotics screen identified *Lacticaseibacillus rhamnosus* HA-114 as a neuroprotective bacterial strain. Transgenics were monitored from the adult stage, scored daily for paralysis. (a) $FUS^{S57\Delta}$ worms fed with *L. helveticus* R0052, *L. paracasei* HA-196, *L. plantarum* HA-119 or *L. casei* L26 have the same paralysis rate as the worms fed with OP50, while worms fed with L. rhamnosus HA-114 showed less paralysis. (b) Transgenic animals fed with either, *B. subtilis* R0179, *P. acidilactici* R1001, *L. plantarum* R1012 or *B. breve* HA-129 do not show any differences in paralysis rates when compared to animals fed with OP50. Animals fed with *B. animalis subsp. lactis* B94 showed less paralysis than the ones fed with OP50, but showed more paralysis than animals supplemented with HA-114. (c) HA-114/B94 (1:1) is the only probiotic blend preventing paralysis in $FUS^{S57\Delta}$ worms, at lesser extent than HA-114 alone. (d) Worms fed with blends with higher concentrations of HA-114, compared to OP50, showed less paralysis compared to transgenics expressing mutant FUS fed with OP50 alone. For paralysis (panels a-d) curves were generated and compared using the log-rank (Mantel–Cox) test. Panel a: OP50 n=211; HA-114 n=212; R0052 n=211; HA-196 n=210; HA-119 n=213; L26 n=210; . Panel b: OP50 n=210; HA-114 n=211; R0179 n=210; R1001 n=211; R1012 n=213; HA-129 n=206; B94 n=213. Panel c: OP50 n= 590; HA-114 n=382; HA-119/B94 (1:1) n=351; HA-114/B94 (1:1) n=361; HA-114/HA-119 (1:1) n=359. Panel d: OP50 n=325; HA-114 n=314; OP50/HA-114 (3:1) n= 315; OP50/HA-114 (1:1) n=312; OP50/HA-114 (1:3) n=314.

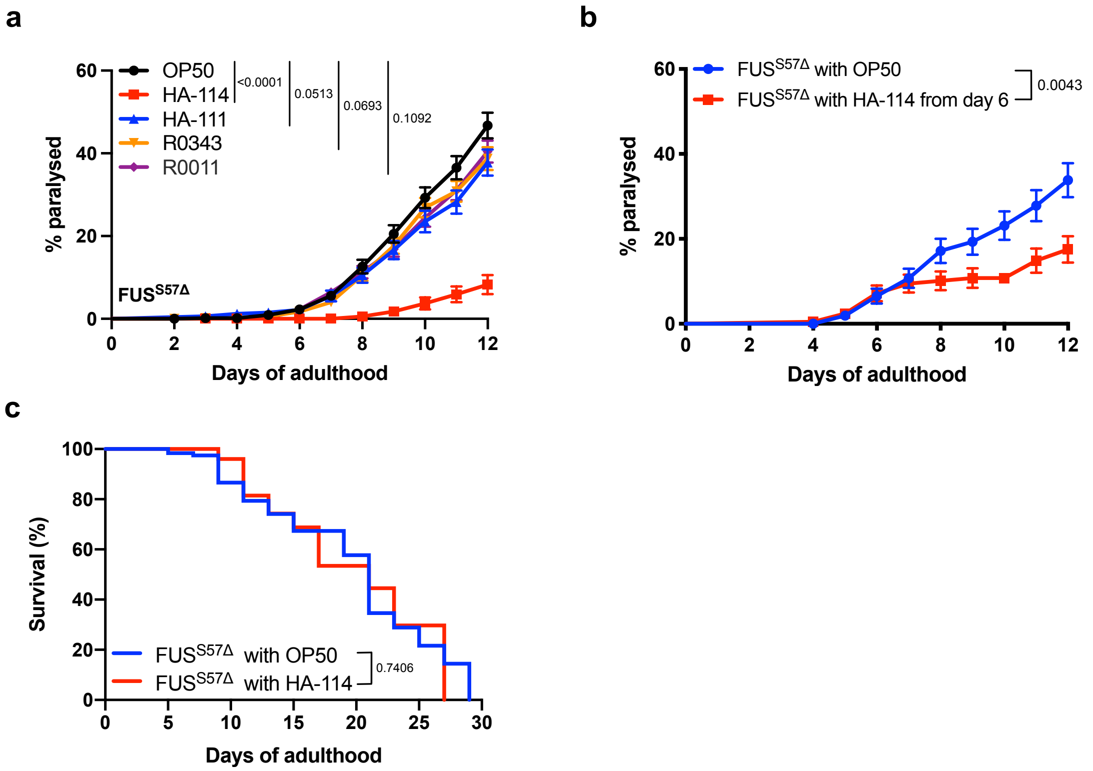

Supplementary figure 2. *Lacticaseibacillus rhamnosus* HA-114 is the only neuroprotective *rhamnosus* strain but does not extend lifespan. Transgenics were monitored from the adult stage, scored daily for paralysis. (a) $FUS^{S57\Delta}$ worms fed with *L. rhamnosu*s strains R0011, R0343 and HA-111 have the same paralysis rate as the worms fed with OP50. (b) Worms fed with HA-114 from day 6 (onset of paralysis) showed less paralysis compared to transgenics expressing mutant FUS fed with OP50. (c) Transgenics were monitored from the adult stage, scored every two days for mortality, and fed with control OP50 or HA-114. Mutant FUS worms fed with HA-114 do not show any lifespan extension when compared to transgenics expressing mutant FUS fed with OP50. For paralysis and lifespan assays (panels a-c) curves were generated and compared using the log-rank (Mantel–Cox) test. Panel a: OP50 n=582; HA-114 n=212; HA-111 n=331; R0343 n=585; R0011 n=588. Panel b: OP50 n=210; HA-114 from day 6 n=206. Panel c: OP50 n=126; HA-114 n=122.

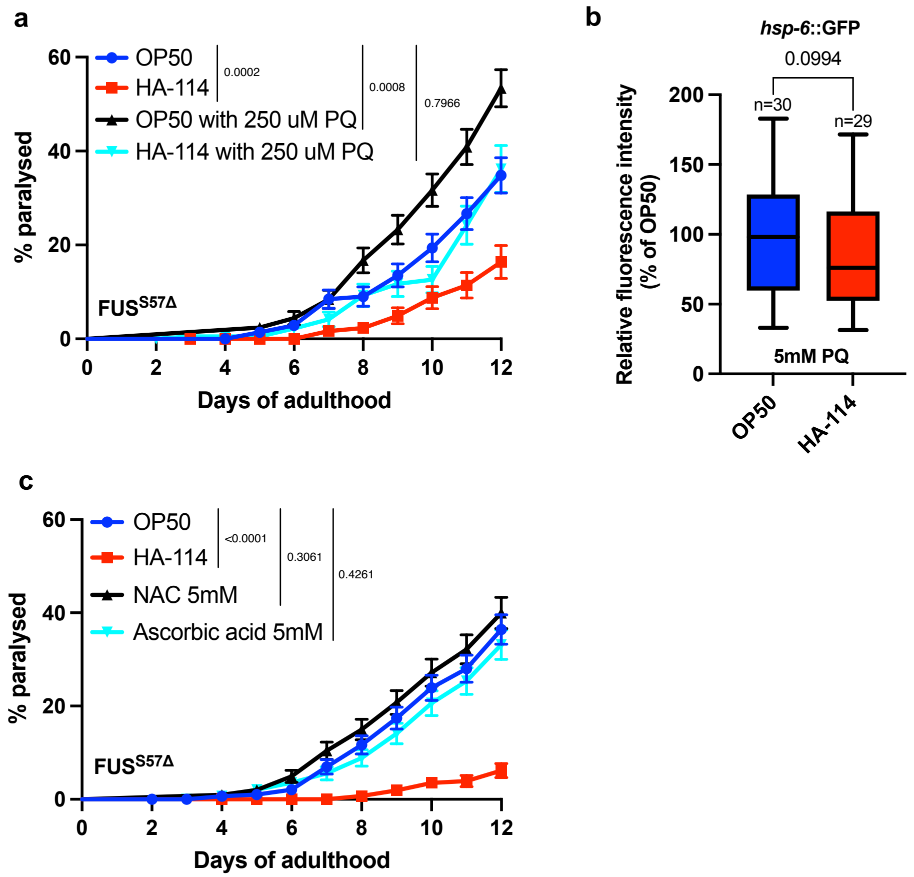

Supplementary figure 3. *Lacticaseibacillus rhamnosus* HA-114 prevents damage from chronic oxidative stress. (a) Transgenics were monitored from the adult stage, scored daily for paralysis. Chronic treatment of 250 μM paraquat increased paralysis in FUS$^{S57\Delta}$ animals. HA-114 supplementation prevents increase of paralysis from paraquat treatment. (b) HA-114 treatment did not decrease hsp-6::GFP signal after acute oxidative stress. (c) Worms treated with either N-acetyl cysteine (NAC) or Ascorbic acid have the same paralysis rate as the worms fed with OP50. For paralysis assays (panels a & c) curves were generated and compared using the log-rank (Mantel–Cox) test. Panel a: OP50 n=216; HA-114 n=196; OP50 with 250μM Paraquat n=205; HA-114 with 250μM Paraquat n=199. Panel c: OP50 n=312; HA-114 n=315; NAC 5mM n=310; Ascorbic acid 5mM n=303. For fluorescence quantification (panel c), an unpaired

t-test was performed, and *n* are indicated in the figure. For boxplots, minimum, first quartile, median, third quartile, and maximum are shown.

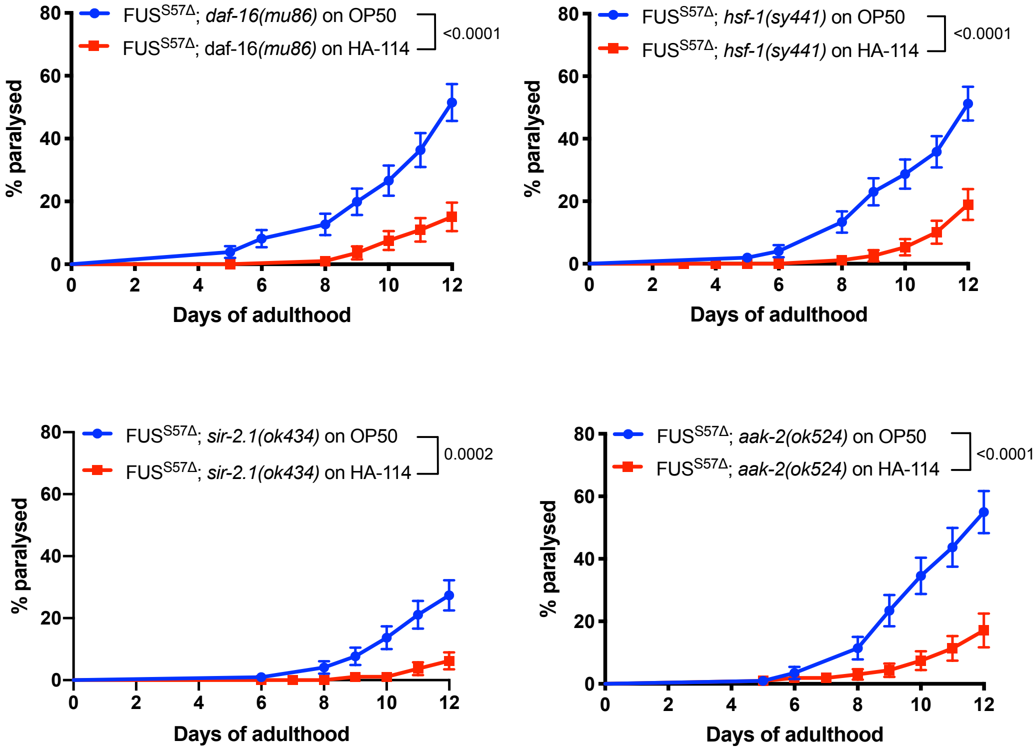

Supplementary figure 4. HA-114 does not require classic stress and metabolic pathways in FUS$^{S57\Delta}$ worms for neuroprotection. Transgenics were monitored from the adult stage, scored daily for paralysis. Neuroprotection provided by *L. rhamnosus* HA-114 was unaffected by *daf-16(mu86)* deletion, nor by *hsf-1(sy441)* point mutation. Both *sir-2.1* and *aak-2* genes are not required for neuroprotection granted by HA-114 probiotics. For paralysis assays, curves were generated and compared using the log-rank (Mantel–Cox) test. FUS$^{S57\Delta}$; *daf-16(mu86)* on OP50 n=103; FUS$^{S57\Delta}$; *daf-16(mu86)* on HA-114 n=106; FUS$^{S57\Delta}$; *hsf-1(sy441)* on OP50 n=103; FUS$^{S57\Delta}$; *hsf-1(sy441)* on HA-114 n=105; FUS$^{S57\Delta}$; *sir-2.1(ok434)* on OP50 n=101; FUS$^{S57\Delta}$; *sir-2.1(ok434)* on HA-114 n=106; FUS$^{S57\Delta}$; *aak-2 (ok524)* on OP50 n=104; FUS$^{S57\Delta}$; *sir-2.1(ok524)* on HA-114 n=107.

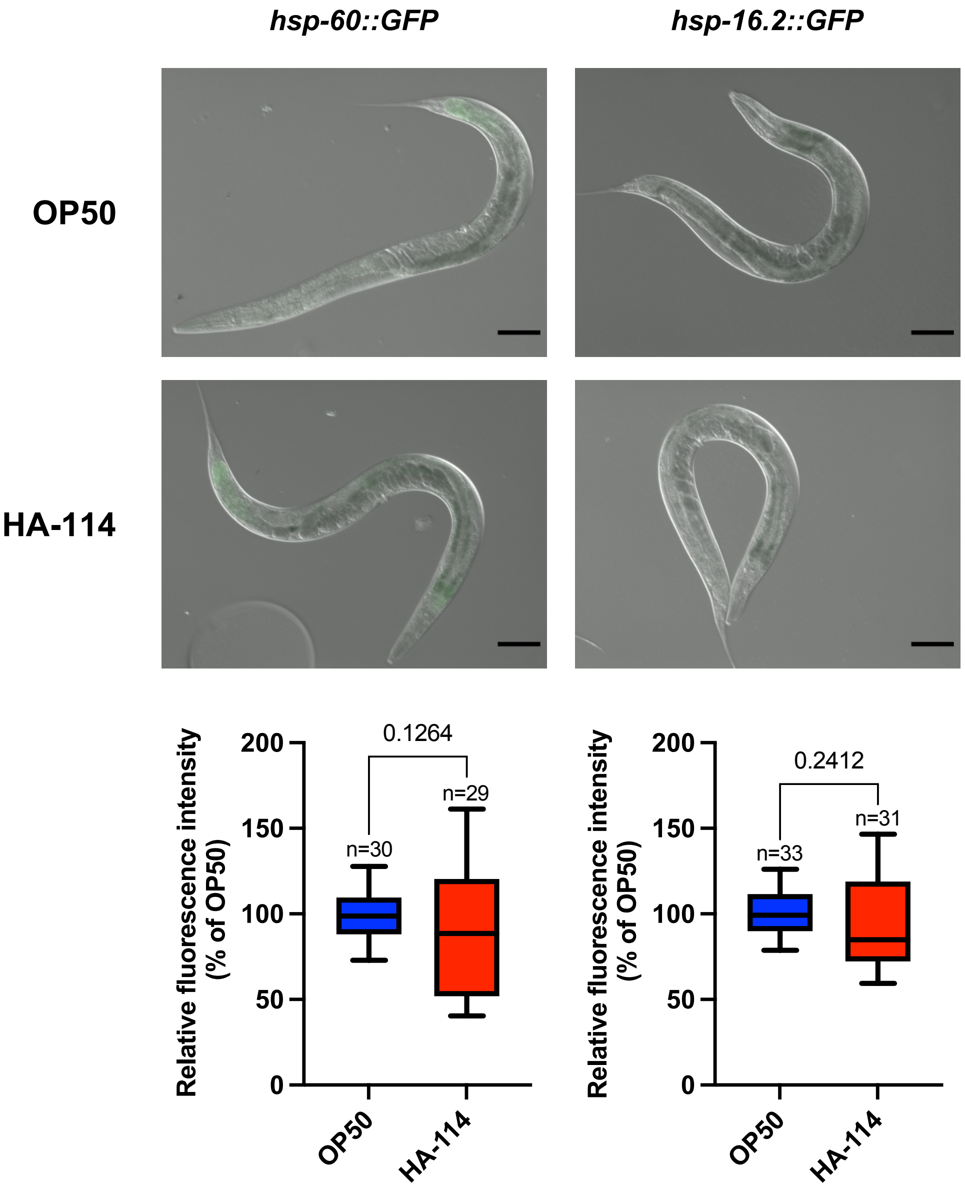

Supplementary figure 5. HA-114 does not activate classic stress pathways in *C. elegans*. HA-114 did not affect GFP expression of key genes of the endoplasmic reticulum unfolded protein response (UPRmito; *hsp-60)* or cytoplasmic unfolded protein response (UPR^{Cyt}; *hsp-16.2)*. For fluorescence quantifications, unpaired t-test were performed, and *n* are indicated in the figure. Scale bar = 100 µm. For boxplots, minimum, first quartile, median, third quartile, and maximum are shown.

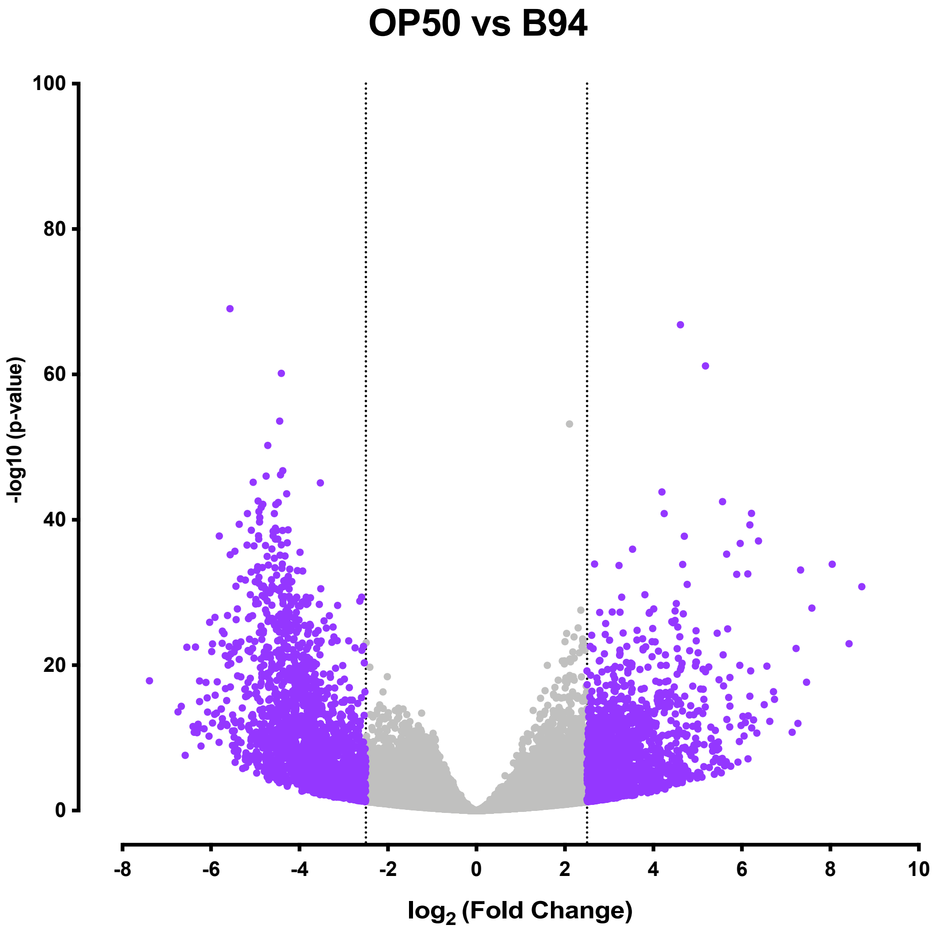

Supplementary figure 6. *Bifidobacterium animalis subsp. lactis* B94 induces differential genes expression in *C. elegans*. Volcano plot of RNA-Seq data of N2 worms fed with *Bifidobacterium animalis subsp. lactis* B94 and compared to worms fed with OP50. The data for all genes were plotted as log2 fold change versus -log10 of the adjusted p-value. Data: GEO accession: GSE189988; SRA study:SRP348888.

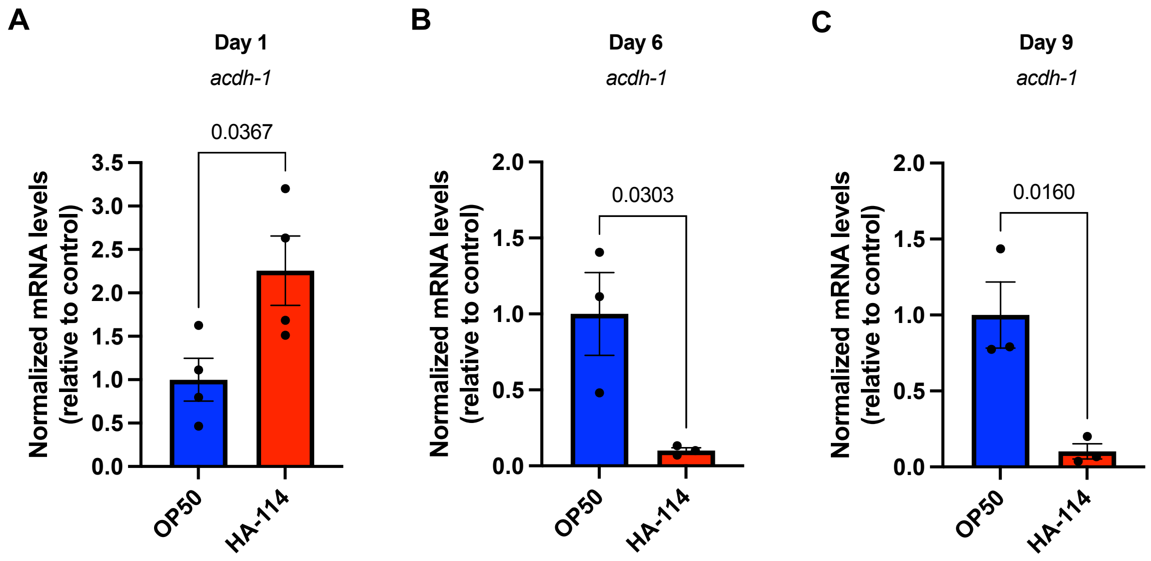

Supplementary figure 7. HA-114 modulate *acdh-1* expression through aging in mutant FUS worms. (a) mRNA expression of *acdh-1* was significantly increased in day 1 $FUS^{S57\Delta}$ worms fed with HA-114 when compared to animals fed with OP50. HA-114 treatment decreased *acdh-1* expression through aging, at day 6 (b) and day 9 (c). For TaqMan assays (panels a-c), an unpaired t-test was performed. Panel a: n=4 per condition. Panel b: n=3 per condition. Panel c: n=3 per condition. Data are presented as mean ± SEM.

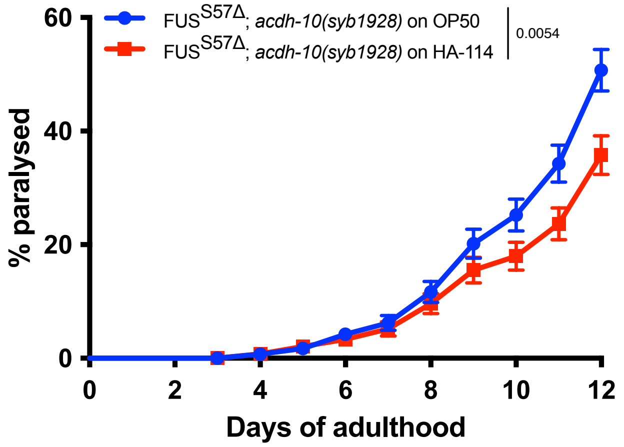

Supplementary figure 8. *acdh-10* is not required for neuroprotection provided by HA-114 in FUS$^{S57\Delta}$ worms. Transgenics were monitored from the adult stage, scored daily for paralysis. Neuroprotection provided by *L. rhamnosus* HA-114 was unaffected by *acdh-10(syb1928)* nonsense mutation. Curves were generated and compared using the log-rank (Mantel–Cox) test. FUS$^{S57\Delta}$; *acdh-10(syb1928)* on OP50 n=419; FUS$^{S57\Delta}$; *acdh-10(syb1928)* on HA-114 n=421.

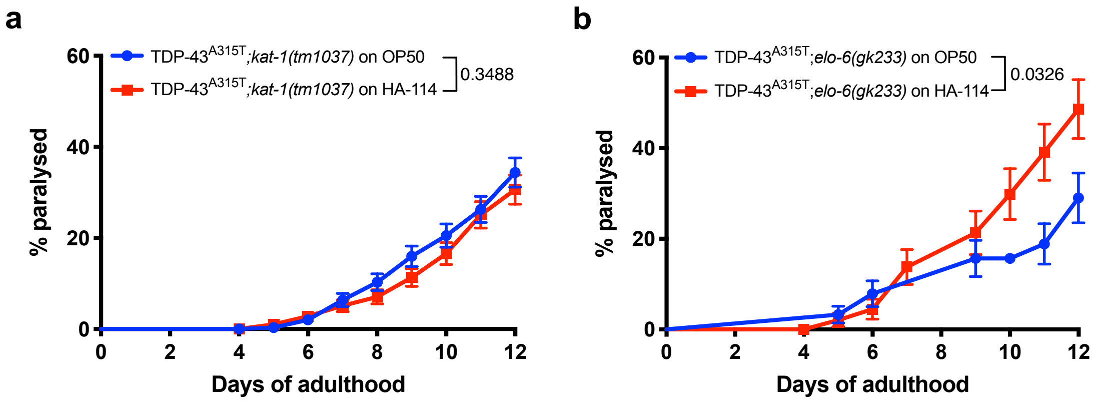

Supplementary figure 9. ***kat-1*** **and** ***elo-6*** **are essential for neuroprotection provided by HA-114 in TDP-43^{A315T} ALS model.** Transgenics were monitored from the adult stage, scored daily for paralysis. Deletion of *kat-1* (a) and *elo-6* (b) altered the beneficial effect of HA-114. For paralysis assays, curves were generated and compared using the log-rank (Mantel–Cox) test. Panel a: TDP-43^{A315T}; *kat-1(tm1037)* on OP50 n=308; TDP-43^{A315T}; *kat-1(tm1037)* on HA-114 n=295. Panel b: TDP-43^{A315T}; *elo-6(gk233)* on OP50 n=92; TDP-43^{A315T}; *elo-6(gk233)* on HA-114 n=102.

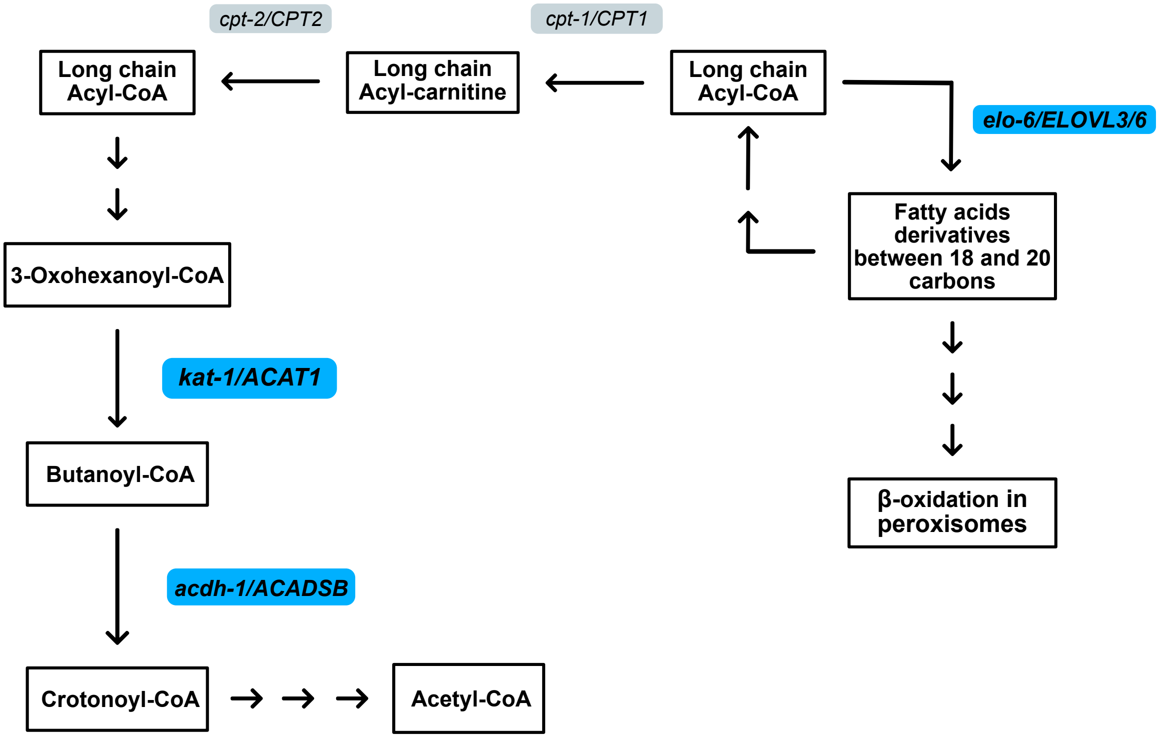

Supplementary figure 10. Simplified pathway of fatty acids metabolism and β-oxidation in *C. elegans* and *H. sapiens*. Enzymes names are enclosed in rounded rectangles. *acdh-1/ACADSB*: Acyl CoA dehydrogenase; *cpt-1/CPT1*: carnitine palmitoyl transferase 1; *cpt-2/CPT2* : carnitine palmitoyl transferase 2; *elo-6/ELOVL3/6:* fatty acid elongase 3/6; *kat-1/ACAT1*: 3-Ketoacyl-coA thiolase.

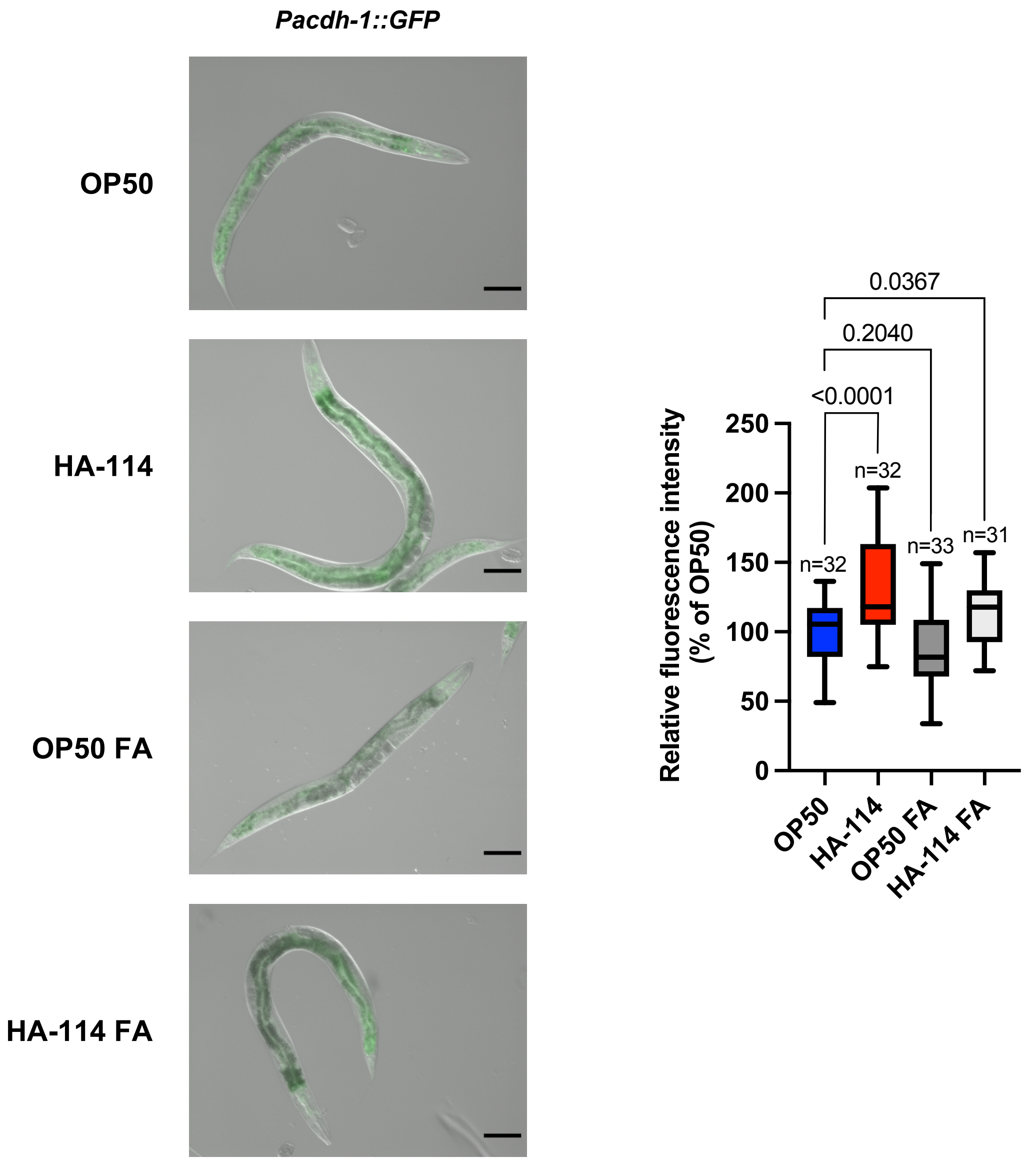

Supplementary figure 11. *acdh-1* is activated by HA-114' fatty acids. Worms were fed with either OP50, HA-114 or a mix of OP50 and fatty acids, extracted from OP50 or HA-114, for 24h before analysis. Fatty acids from OP50 did not affect GFP expression of the *Pacdh-1::GFP* reporter strain, while HA-114' fatty acids were enough to increased GFP signal. For fluorescence quantification, one-way ANOVA were performed, and *n* are indicated in the figure. Scale bar = 100 µm. For boxplots, minimum, first quartile, median, third quartile, and maximum are shown.

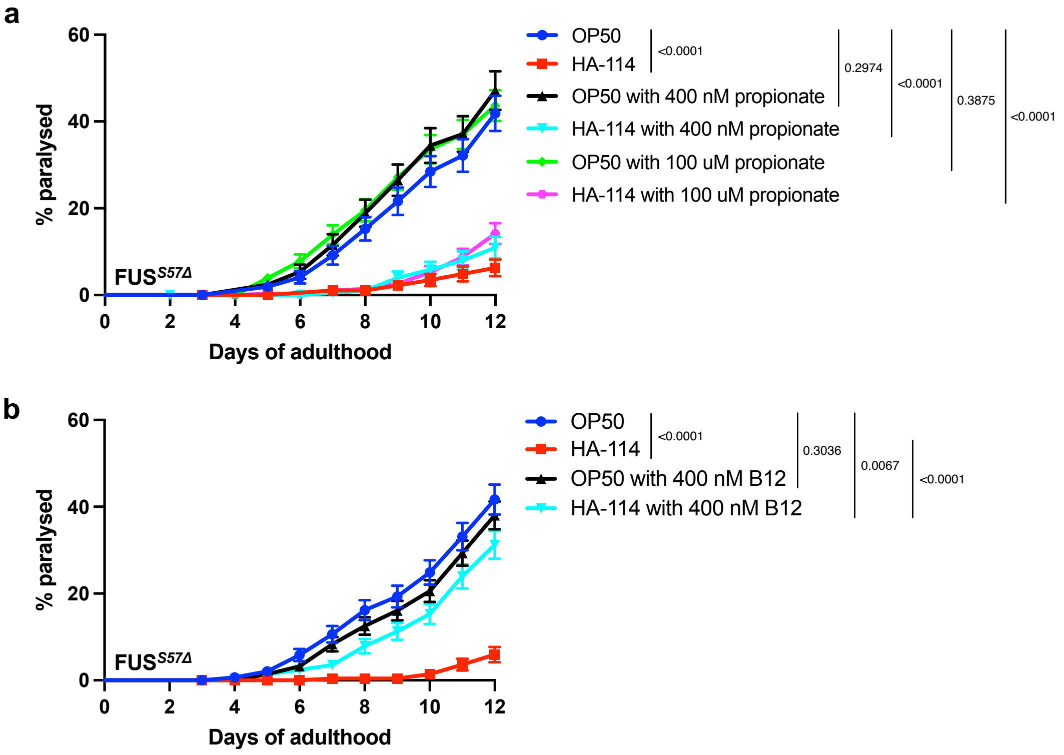

Supplementary figure 12. Modulating *acdh-1* pathway partially affects HA-114 neuroprotective effect. Transgenics were monitored from the adult stage, scored daily for paralysis. (a) Supplementation of propionate does not rescue paralysis phenotype in FUS$^{S57\Delta}$ worms. Combination of HA-114 and propionate does not cause propionate toxicity in these animals. (b) Vitamin B12 supplementation prevent HA-114 to have its neuroprotective effect, but does not exacerbate paralysis phenotype in OP50 fed worms. For paralysis assays, curves were generated and compared using the log-rank (Mantel–Cox) test. Panel a: OP50 n=211; HA-114 n=208; OP50 with 400nM propionate n=212; HA-114 with 400nM propionate n=211; OP50 with 100uM propionate n=317; HA-114 with 100uM propionate n=290. Panel c: OP50 n=314; HA-114 n=262; OP50 with 400nM B12 n=300; HA-114 with 400nM B12 n=299.

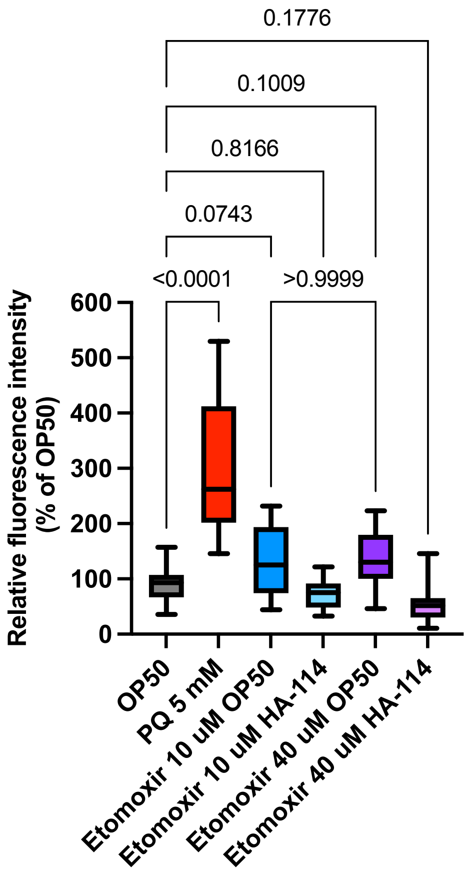

Supplementary figure 13. Etomoxir does not cause oxidative stress in *C. elegans*. Transgenics were treated with Etomoxir for 24h before analysis. Etomoxir does not significatively increase *hsp-6::GFP* signal, on either OP50 or HA-114 diet. For fluorescence quantification, one-way ANOVA was performed. OP50 n=30, PQ 5mM n=27, Etomoxir 10uM on OP50 n=29, Etomoxir 10uM on HA-114 n=29, Etomoxir 40uM on OP50 n=28, Etomoxir 40uM on HA-114 n=29. For boxplots, minimum, first quartile, median, third quartile, and maximum are shown.

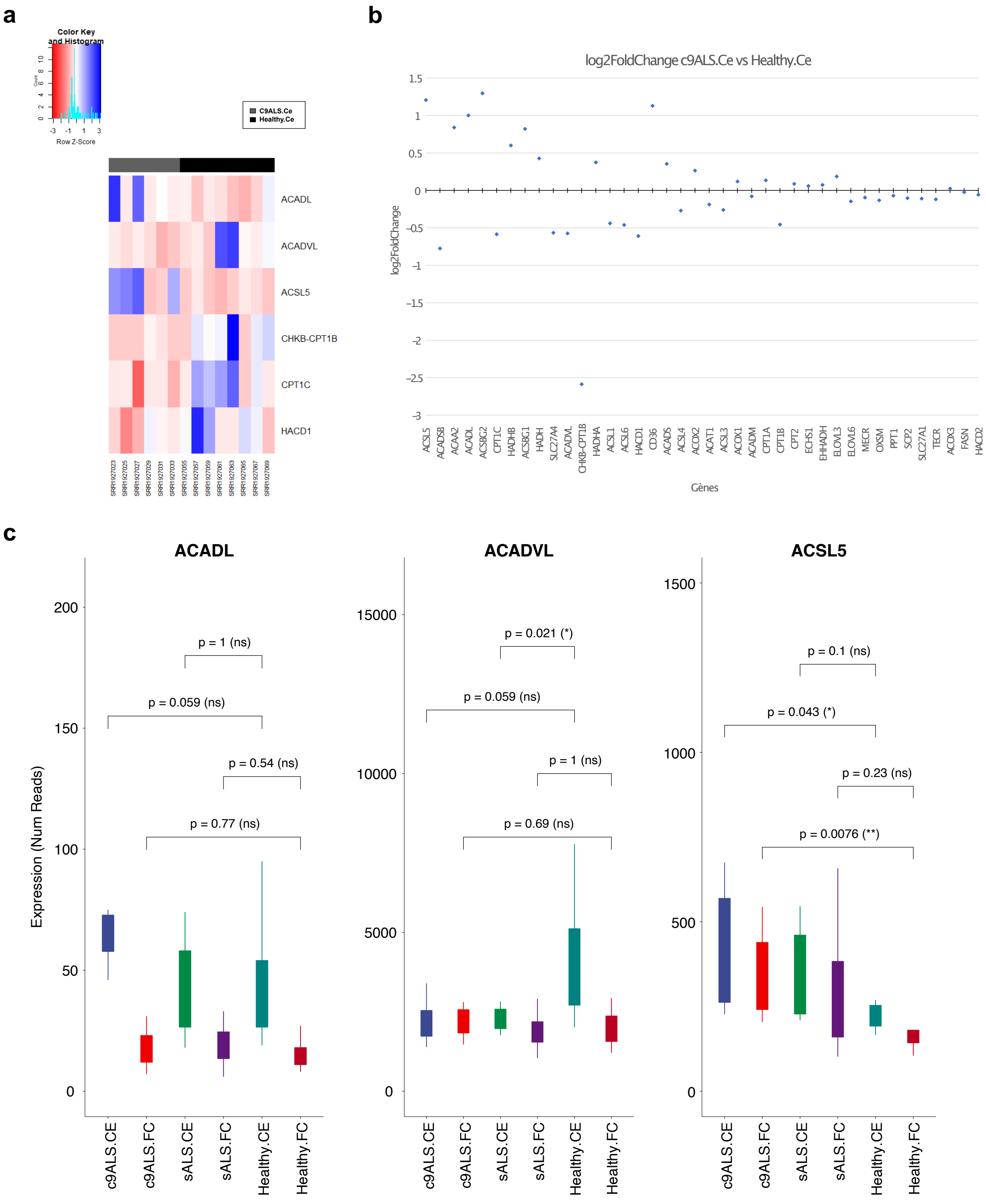
a
Color Key and Histogram
Count
Row Z-Score
C9ALS.Ce
Healthy.Ce
ACADL
ACADVL
ACSL5
CHKB-CPT1B
CPT1C
HACD1
b
log2FoldChange c9ALS.Ce vs Healthy.Ce
log2FoldChange
Gènes
ACSL5
ACADSB
ACAA2
ACADL
ACSBG2
CPT1C
HADHB
ACSBG1
HADH
SLC27A4
ACADVL
CHKB-CPT1B
HADHA
ACSL1
ACSL6
HACD1
CD36
ACADS
ACSL4
ACOX2
ACAT1
ACSL3
ACOX1
ACADM
CPT1A
CPT1B
CPT2
ECHS1
EHHADH
ELOVL3
ELOVL6
MECR
OXSM
PPT1
SCP2
SLC27A1
TECR
ACOX3
FASN
HACD2
c
ACADL
ACADVL
ACSL5
Expression (Num Reads)
p = 1 (ns)
p = 0.059 (ns)
p = 0.54 (ns)
p = 0.77 (ns)
p = 0.021 (*)
p = 0.059 (ns)
p = 1 (ns)
p = 0.69 (ns)
p = 0.1 (ns)
p = 0.043 (*)
p = 0.23 (ns)
p = 0.0076 (**)
c9ALS.CE
c9ALS.FC
sALS.CE
sALS.FC
Healthy.CE
Healthy.FC

Supplementary figure 14: Genes associated with β-oxidation are differentially expressed in the cerebellum and the frontal cortex of c9ALS patients. (a) Differentially expressed genes associated with the β-oxidation pathway between *C9orf72* ALS patients and healthy individuals were plotted as heatmap. Red indicates low relative expression and blue indicates high relative expression. (b) Log2 Fold change of differentially expressed genes associated with the β-oxidation pathway between *C9orf72* ALS patients and healthy individuals. (c) *ACADL* expression is upregulated in the cerebellum of c9ALS and *ACADVL* is downregulated in c9ALS and sALS cerebellum. Interestingly, *ACSL5* expression is upregulated in the cerebellum and the frontal cortex of c9ALS. Data: GEO accession: GSM1642314; SRA study: SRP056477. For boxplots, minimum, first quartile, third quartile, and maximum are shown.

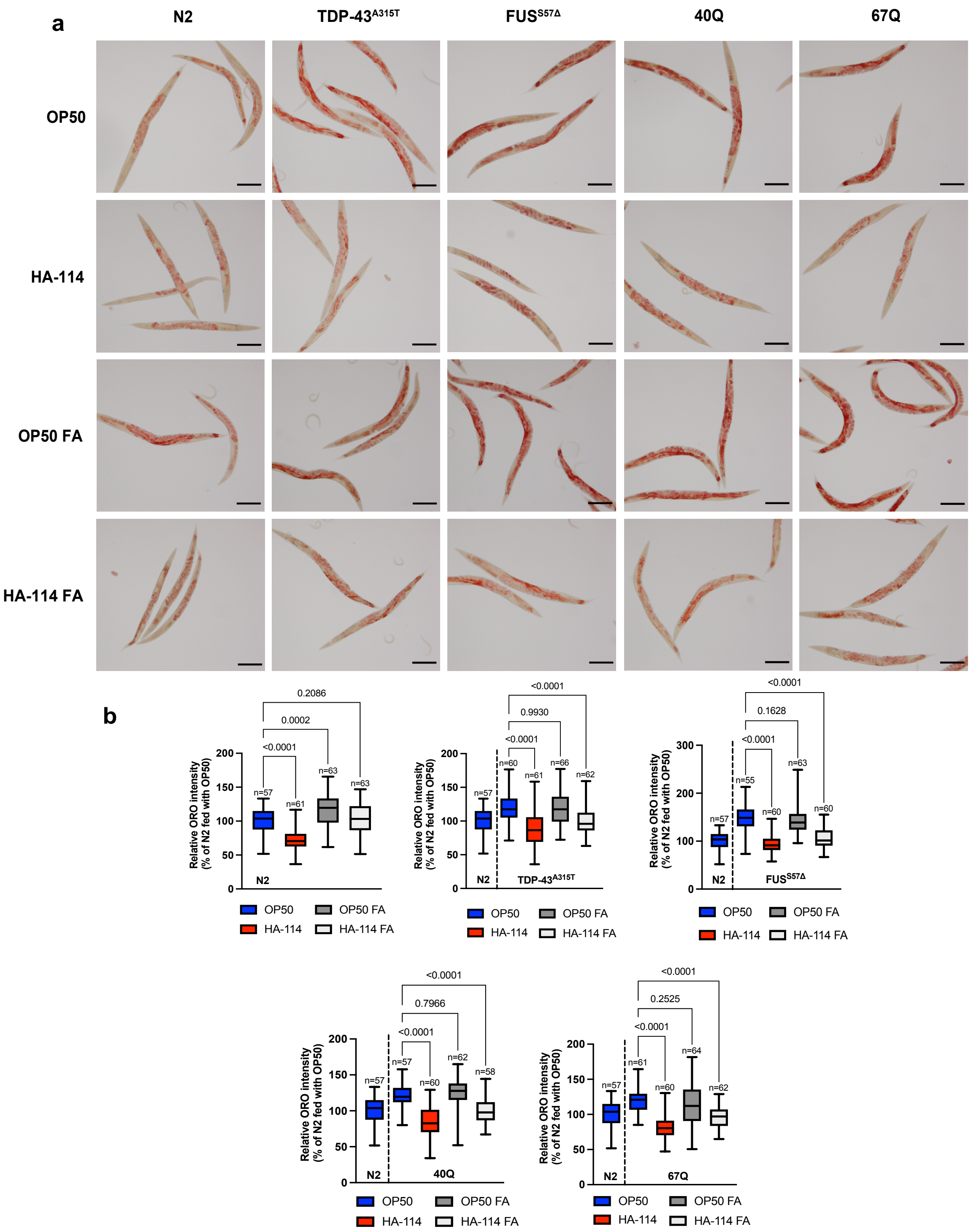
a
N2
TDP-43A315T
FUSS57Δ
40Q
67Q
OP50
HA-114
OP50 FA
HA-114 FA
b
Relative ORO intensity (% of N2 fed with OP50)
0.2086
0.0002
<0.0001
n=57
n=61
n=63
n=63
N2
<0.0001
0.9930
<0.0001
n=57
n=60
n=61
n=66
n=62
TDP-43A315T
<0.0001
0.1628
<0.0001
n=57
n=55
n=60
n=63
n=60
FUSS57Δ
<0.0001
0.7966
<0.0001
n=57
n=57
n=60
n=62
n=58
40Q
<0.0001
0.2525
<0.0001
n=57
n=61
n=60
n=64
n=62
67Q
OP50
OP50 FA
HA-114
HA-114 FA

Supplementary figure 15. Fatty acids from HA-114 modulate lipid accumulation in worm age-dependent neurodegeneration models. (a) Representative images of worms fed with OP50, *L. rhamnosus* HA-114, fatty acids from OP50 or fatty acids from HA-114 and stained with Oil Red O. N2 worms show a basal level of lipid accumulation. (b) Quantification of Oil Red O show same levels of fat accumulation in worms expressing $FUS^{S57\Delta}$, $TDP\text{-}43^{A315T}$, and polyQ expansion (40Q and 67Q) when fed with either OP50 or fatty acid extract from OP50. Worms fed with fatty acids from HA-114 showed a significant decrease of lipid accumulation. For the Oil Red O quantification graphs (panel b), one-way ANOVA were performed and *n* are indicated in the figure. Scale bar = 100 µm. For boxplots, minimum, first quartile, median, third quartile, and maximum are shown.

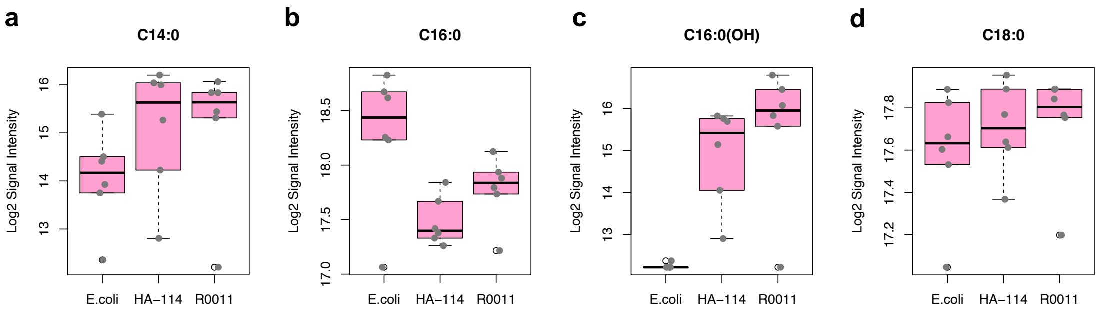

Supplementary figure 16. Free fatty acids (FFA) annotated from OP50, HA-114 and R0011 bacterial strains. Log2 signal intensity from (a) C14:0 myristic acid (HA-114 vs OP50 p=0.1618594; R0011 vs OP50 p=0.1730919; HA-114 vs R0011 p=0.8966804), (b) C16:0 palmitic acid (HA-114 vs OP50 p=0.01408153; R0011 vs OP50 p=0.11738097; HA-114 vs R0011 p=0.0840674), (c) C16:0(OH) hydroxypalmitic acid (HA-114 vs OP50 p=0.0002694804; R0011 vs OP50 p=0.0007397631; HA-114 vs R0011 p=0.391468), (d) C18:0 stearic acid (HA-114 vs OP50 p=0.5338358; R0011 vs OP50 p=0.4425402; HA-114 vs R0011 p=0.901340). For lipidomics analysis, n=6 for each bacterial strain. Raw data and untargeted lipidomic results analysis are available in Supplementary Data 1 and 2. For boxplots, minimum, first quartile, median, third quartile, maximum and each data point are shown.

Gene	log2 (Fold Change)	-log10 (p-value)	Gene	log2 (Fold Change)	-log10 (p-value)
comt-4	9.87862663394132	99.7453109	*T07G12.5*	6.60805071963761	27.6992845
fmo-2	9.54793672826199	43.6786685	*Y40B10A.10*	6.55437120438937	9.83497546
col-37	8.09882592083367	23.6043977	*clec-101*	6.53234125111637	15.4291571
col-51	7.99974875772954	16.5212658	*cyp-14A4*	6.47819223000637	17.2524927
scl-12	7.77678012343102	13.627124	*clec-60*	6.46941490655043	21.5608881
ZK355.8	7.72806356442673	24.2158052	*grl-23*	6.45038706815409	16.1948104
nlp-39	7.70628621197532	41.4471206	*W02D7.11*	6.40679336017293	10.5975775
scl-13	7.64237045387068	12.5438689	*col-35*	6.38208805437359	16.3087854
mtl-1	7.63840864172396	73.3528053	*col-36*	6.36194387462439	14.6750336
C54C8.12	7.58426311128764	30.6444151	*Y57E12B.11*	6.35114125562006	8.84129742
spp-20	7.57758760470988	21.0188592	*Y73B6BL.37*	6.33850300181293	14.6128333
col-2	7.45257342787137	19.8318924	*col-108*	6.29215024624157	12.7314131
col-185	7.32752273324769	16.5394412	*Y50E8A.19*	6.24646404668113	11.6861425
ZK355.3	7.27234875958693	18.9184428	*lipl-3*	6.1771018660647	22.7659395
col-50	7.09906776970553	13.0342336	*M6.11*	6.13593697212128	9.97317931
scl-11	6.92409948853672	9.99911968	*ttr-19*	6.06718786890319	8.88081259
col-85	6.90911404977131	17.2538545	*Y57E12B.4*	6.01948735106341	13.5724462
col-183	6.83294309056956	28.6669326	*grl-20*	5.99933755902934	12.3038485
grl-25	6.75068617466407	16.5134975	*smf-3*	5.98431945049091	37.4449756
col-44	6.69878248210145	18.0031516	*C13A2.12*	5.95549751109163	9.2819474
cut-1	6.65779636590876	13.9896961	*gst-19*	5.94282889293229	12.789848
col-40	6.64994189406571	19.6592105	*F20E11.17*	5.93693818626582	11.7431324
col-102	6.63923155878408	13.4238371	*col-84*	5.88220338126241	11.4107272
col-123	6.63889601447437	13.4320701	*col-164*	5.88053302510974	9.39537277
F57F4.2	6.61010243445913	12.0319099	*col-114*	5.83481063285872	13.0582472

Supplementary Table 1: List of upregulated genes in N2 worms fed with HA-114 (compared to OP50)

Gene	log2 (Fold Change)	-log10 (p-value)	Gene	log2 (Fold Change)	-log10 (p-value)
**D1014.6**	-6.58130429443456	7.59723595	*H31G24.1*	-3.36110423381441	4.99501892
cyp-35A3	-5.26971806442959	9.8943547	*T26C11.3*	-3.35994858567097	2.10186277
T20H9.6	-4.77232062732769	4.86362304	*F49F1.8*	-3.34030650862598	2.68831392
Y38H6C.23	-4.39581701915455	5.76033539	*F36F12.3*	-3.32218550668426	2.66452973
acdh-1	-4.27824066606366	7.77503269	*mltn-3*	-3.30974507153072	4.11888428
T24D5.5	-4.27376263570936	4.90651812	*K08F11.6*	-3.29922845314491	11.3203483
nspe-7	-3.95673616454527	8.85512815	*clec-247*	-3.28672949400989	1.90351031
Y32G9A.11	-3.95309359571931	2.82279758	*cutl-7*	-3.27955819396815	8.02778931
cyp-35A4	-3.92865080818664	8.17889876	*ferl-1*	-3.27206188596272	2.3889255
F02H6.1	-3.91853525687342	10.7321835	*C04G6.6*	-3.24534306851372	5.46675923
F32A11.5	-3.84827550994329	6.88243496	*Y49E10.26*	-3.22848634462104	9.89937162
mboa-5	-3.78653834654188	2.90612659	*C44C10.5*	-3.20163254992974	4.60563122
B0554.4	-3.77878513898639	4.47307032	*dot-1.5*	-3.16513176281623	4.52525103
C09G12.17	-3.74519597364298	2.56419407	*B0250.11*	-3.15152606307824	1.95390021
oac-42	-3.7161674910615	3.10876041	*Y105C5B.1420*	-3.14592437731143	1.79352711
F18A12.7	-3.66831246728887	9.02666446	*C04E12.10*	-3.1214471921964	2.75762216
ZC84.1	-3.63205853382885	5.36165306	*Y47G6A.15*	-3.11295496938544	12.9674724
B0554.2	-3.61046784466151	2.26374503	*bath-23*	-3.08795367996044	3.29868589
C04G6.13	-3.57846422518453	2.3533466	*cutl-6*	-3.0868579702075	5.95140556
nas-15	-3.55399028351787	9.5448918	*F52G3.3*	-3.08053730032058	1.78710001
ZK218.1	-3.50723405639355	2.38502544	*Y49E10.30*	-3.05917236626404	3.62565075
F16H6.4	-3.49646819274977	2.20961034	*Y44A6B.3*	-3.04303932102096	3.02291594
col-148	-3.473671185875	5.30595715	*gcy-18*	-3.02977374072766	4.67264013
C32H11.6	-3.41679538726944	3.1785791	*C06A1.7*	-3.02194395846066	1.80489206
F22H10.4	-3.38583404474747	2.33439917	*Y20F4.8*	-3.01879749443978	3.73066234

Supplementary Table 2: List of downregulated genes in N2 worms fed with HA-114 (compared to OP50)

Gene	log2 (Fold Change)	-log10 (p-value)	Gene	log2 (Fold Change)	-log10 (p-value)
fmo-2	8.70956706	30.80558353	*cnc-11*	6.07894161	12.10115523
ilys-3	8.4244723	22.97385399	*C18H7.1*	6.04949025	10.2658901
lipl-3	8.04282975	33.90639009	*fbxa-24*	6.0220537	12.96771729
C54C8.12	7.58490125	27.87428669	*Y73B6BL.37*	5.97947893	11.71166751
ilys-2	7.46101799	17.66153356	*hsp-16.41*	5.96168784	36.75574214
nlp-39	7.32972011	33.10588398	*F49H6.5*	5.95512499	19.99147225
W02D7.11	7.26688977	11.98340288	*C08F1.8*	5.94529945	9.509269995
clec-60	7.2261522	22.32029752	*cyp-33C1*	5.91201039	6.673025068
clx-1	7.13852843	10.76526756	*acs-2*	5.88214796	32.50578365
F40G12.5	6.73873737	15.29875601	*Y46H3A.5*	5.74813101	6.115619855
F57G4.11	6.71539844	16.36843041	*Y53G8AM.5*	5.73195427	18.30334685
W08A12.4	6.63368495	12.2906029	*H39E23.3*	5.73052693	14.38396695
T22F3.11	6.56892797	19.87494156	*ZK971.1*	5.72632025	11.51830638
spp-20	6.50670072	14.56788288	*F07C4.12*	5.70130533	15.5649083
tts-1	6.37615852	37.09018362	*E02H4.4*	5.67989988	25.00699877
rab-11.2	6.34038243	10.66537805	*fbxa-165*	5.66322171	12.54398774
dod-21	6.26681417	12.48657479	*Y57E12B.11*	5.65483204	6.497716389
lys-10	6.22140365	11.37483603	*W03F9.4*	5.65472041	35.29341093
mtl-1	6.22053905	40.89787225	*Y60C6A.2*	5.64772622	6.738652939
F46A8.13	6.19936449	19.22317505	*scl-11*	5.60865357	6.147619693
thn-1	6.18271149	15.73992935	*T07G12.5*	5.58558481	17.13924825
hsp-16.2	6.18204413	39.29514003	*srg-31*	5.57982942	21.43650861
F08H9.3	6.14357275	13.05352608	*sodh-1*	5.56438661	42.50494404
irld-36	6.14111929	7.137245121	*C44H9.2*	5.55996121	7.192404706
ugt-18	6.1360785	32.57612213	*ins-8*	5.53446772	5.211344066

Supplementary Table 3: List of upregulated genes in N2 worms fed with B94 (compared to OP50)

Gene	log2 (Fold Change)	-log10 (p-value)	Gene	log2 (Fold Change)	-log10 (p-value)
fipr-18	-7.3899051	17.8629289	*ssp-19*	-5.9031358	15.8683656
C16C8.19	-6.7450299	13.5896424	*K02B12.6*	-5.8777989	13.4207461
F49C5.11	-6.6720748	14.3588193	*fipr-20*	-5.8562662	17.7295597
D1014.6	-6.5833517	7.60330179	*fis-1*	-5.8136158	9.37802511
Y39B6A.9	-6.5445814	22.4913188	*bli-1*	-5.8095208	37.7755119
msp-71	-6.4031816	11.5842834	*ZK970.8*	-5.780491	11.5692106
R01E6.5	-6.3793551	10.775615	*K08C9.1*	-5.768231	13.9929598
T16G12.10	-6.349873	22.5070685	*scl-7*	-5.763782	13.9937634
ZK1025.3	-6.310217	10.7349212	*C05B5.11*	-5.7464197	23.005554
K12H6.8	-6.2913412	11.8767872	*F25C8.1*	-5.741145	12.6612795
T26E3.6	-6.2636633	11.3583125	*col-138*	-5.7397982	24.6828362
F46B6.13	-6.255397	15.0117974	*R09H10.6*	-5.7367796	12.0214734
F26D10.13	-6.2547035	17.8234353	*rol-1*	-5.7045872	24.3361006
Y102E9.6	-6.2251704	8.86862954	*C48B4.12*	-5.6782743	21.3184496
R03H10.4	-6.1527173	11.2609968	*C09G12.5*	-5.671836	16.7723887
clec-99	-6.1141736	17.631972	*col-49*	-5.6509951	11.8762189
fipr-28	-6.0860342	15.5071309	*M176.10*	-5.6425164	15.1383091
F43C11.2	-6.0760143	13.5530852	*C16C8.7*	-5.6337892	11.7216452
fipr-19	-6.0407717	10.2289594	*try-8*	-5.6258549	26.8288941
tag-329	-6.0305397	25.8986167	*K12H6.4*	-5.6134512	20.0022469
clec-207	-5.9805215	21.875854	*clec-219*	-5.6047092	21.0832096
clec-208	-5.9653792	22.9368451	*F18E9.7*	-5.5864444	22.5636954
cutl-7	-5.9623714	12.0496394	*F35E12.5*	-5.5700749	69.0611886
R11G10.4	-5.9192587	13.2498415	*col-110*	-5.5656775	35.1928499
bli-2	-5.9096492	26.5864057	*K03D3.5*	-5.559995	17.0099999

Supplementary Table 4: List of downregulated genes in N2 worms fed with B94 (compared to OP50)

Gene	Forward	Reverse
kat-1	GCCCGTACACCTATTGGATC	CTTCTGGTCCGATATTTCCA
acdh-1	TCCAGCTAATGGGTGTTCATG	GTGCCTGTGGTGTTTCTAC
elo-6	GACTCACATCGCATTTCTGG	GAGACTTGTACCTTGTACCCA
aak-2	CCTGGCAACACCATAAGCTG	CAGGGTTCCACAAAGAAGTG
daf-16	TCTCCTCCTGTGTGCCTTCC	CTTTATCCTCTTCTTGGCTCCG
sir-2.1	ACTTCTGGTCGTCTTCTTCG	CTTTCGCAATGTGTCTCGTG
hsf-1	AATCCTCGGCTCCATCATAATTCG	CAGCCGCAACAAGACTATTCG
acs-20	GAGGAACCTCTACACCATAAACAG	CCTTCTGATCGTATGTACGTCTC
acdh-10	ATCTTCCAAATCTACGAGGG	TTCAACAATGAGGGCAGTCT
Human *TARDBP*	GTCTCTTTGTGGAGAGGACTTGATC	GGTTTGGCTCCCTCTGCATG
Human *FUS*	ATGGCCTCAAACGATTATAC	GATTATAGCTTTGCTGCTGT

Supplementary Table 5: List of oligo sequences used for genotyping
