# Supplementary material for: Fatty acids derived from the probiotic Lacticaseibacillus rhamnosus HA-114 suppress age-dependent neurodegeneration
Source: Commun Biol. 2022 Dec 7;5:1340. doi: 10.1038/s42003-022-04295-8 (PMC9729297; doi:10.1038/s42003-022-04295-8)
Supplement: Supplementary file 3 — Description of Additional Supplementary Files [file 42003_2022_4295_MOESM3_ESM.pdf]

Description of Additional Supplementary Files

File name: Supplementary Data 1

Description: The raw data from the lipidomics

File name: Supplementary Data 2

Description: The Untargeted lipidomics results

File name: Supplementary Data 3

Description: The source data behind the graphs in the paper
