# Supplementary material for: Fatty acids derived from the probiotic Lacticaseibacillus rhamnosus HA-114 suppress age-dependent neurodegeneration
Source: Commun Biol. 2022 Dec 7;5:1340. doi: 10.1038/s42003-022-04295-8 (PMC9729297; doi:10.1038/s42003-022-04295-8)
Supplement: Supplementary file 7 — Reporting Summary [file 42003_2022_4295_MOESM7_ESM.pdf]

nature portfolio

Corresponding author(s): J Alex Parker

Last updated by author(s): Nov 21, 2022

Reporting Summary

Nature Portfolio wishes to improve the reproducibility of the work that we publish. This form provides structure for consistency and transparency in reporting. For further information on Nature Portfolio policies, see our Editorial Policies and the Editorial Policy Checklist.

Statistics

For all statistical analyses, confirm that the following items are present in the figure legend, table legend, main text, or Methods section.

n/a	Confirmed	
☐	☒	The exact sample size (*n*) for each experimental group/condition, given as a discrete number and unit of measurement
☐	☒	A statement on whether measurements were taken from distinct samples or whether the same sample was measured repeatedly
☐	☒	The statistical test(s) used AND whether they are one- or two-sided<br>*Only common tests should be described solely by name; describe more complex techniques in the Methods section.*
☒	☐	A description of all covariates tested
☐	☒	A description of any assumptions or corrections, such as tests of normality and adjustment for multiple comparisons
☐	☒	A full description of the statistical parameters including central tendency (e.g. means) or other basic estimates (e.g. regression coefficient) AND variation (e.g. standard deviation) or associated estimates of uncertainty (e.g. confidence intervals)
☐	☒	For null hypothesis testing, the test statistic (e.g. *F*, *t*, *r*) with confidence intervals, effect sizes, degrees of freedom and *P* value noted<br>*Give P values as exact values whenever suitable.*
☒	☐	For Bayesian analysis, information on the choice of priors and Markov chain Monte Carlo settings
☒	☐	For hierarchical and complex designs, identification of the appropriate level for tests and full reporting of outcomes
☒	☐	Estimates of effect sizes (e.g. Cohen's *d*, Pearson's *r*), indicating how they were calculated

Our web collection on statistics for biologists contains articles on many of the points above.

Software and code

Policy information about availability of computer code

Data collection	All fluorescence microscopy images were taken using a Zeiss microscope AxioObserver Z1 with AxioVs40 4.8.2.0 software. Brightfield images were taken using an Olympus brightfield microscope with a DP21 microscope digital system.
Data analysis	GraphPad Prism 8.0 was used for statistical analysis and generating graphs, at the exception of bioinformatics analysis, were R was used.<br>Fiji (Image J) was used for image quantitation.<br>RNA-seq analysis from C. elegans: Trimmomatic (version 0.35), STAR (version 2.5.1b), DESeq2 (version 1.6.2)<br>GO-term analysis: PANTHER 11<br>Bioinformatics analysis on RNA-sequencing data from human: HiSAT2, HTSeq-count, Bioconductor R package DESeq2<br>Lipidomics analysis: Mass Hunter B.06.00, R software

For manuscripts utilizing custom algorithms or software that are central to the research but not yet described in published literature, software must be made available to editors and reviewers. We strongly encourage code deposition in a community repository (e.g. GitHub). See the Nature Portfolio guidelines for submitting code & software for further information.

Data

Policy information about availability of data

All manuscripts must include a data availability statement. This statement should provide the following information, where applicable:

- Accession codes, unique identifiers, or web links for publicly available datasets
- A description of any restrictions on data availability
- For clinical datasets or third party data, please ensure that the statement adheres to our policy

The authors declare that all relevant data supporting the findings of this study are available within the paper and its supplementary information files. Lipidomics

data sets (Figure 7 and S16) are available in Supplementary Data 1 & 2. RNA-Seq data generated for this manuscript (Figure 3 and S6) have been deposited on NCBI's Gene Expression Omnibus (GEO) (GSE189988; SRA study SRP348888; https://www.ncbi.nlm.nih.gov/geo/query/acc.cgi?acc=GSE189988). RNA-Seq data used for this manuscript (Figure 4 and S12) are publicly available on NCBI's Gene Expression Omnibus (GEO) (GSM1642314; SRA study: SRP056477; https://www.ncbi.nlm.nih.gov/geo/query/acc.cgi?acc=GSE67196) 60. Source data for all figures are provided with the paper (Figure 1-8 and Figures S1-S16) in Supplementary Data 3. Any remaining raw data will be available from the corresponding author upon reasonable request.

Field-specific reporting

Please select the one below that is the best fit for your research. If you are not sure, read the appropriate sections before making your selection.

☒ Life sciences ☐ Behavioural & social sciences ☐ Ecological, evolutionary & environmental sciences

For a reference copy of the document with all sections, see nature.com/documents/nr-reporting-summary-flat.pdf

Life sciences study design

All studies must disclose on these points even when the disclosure is negative.

Sample size	In our experiments, sample sizes were estimated based on previous literatures and were chosen in order to be able to perform statistical analyses, as is standard in the field (Wormbook as a reference). All n are clearly indicated in figures or figure descriptions, as well as in the data source file.
Data exclusions	In all worms experiences, worms that crawled off the plate were not included in the data. Bagged worms were excluded from lifespan analysis. Dead worms were excluded from paralysis assay. Prism 8.0 outlier tool was used in order to assess data exclusion.
Replication	All attempts at replication were successful
Randomization	Worms were chosen unbiasedly for experimental analysis to ensure randomization.
Blinding	For some experiments, plates were prepared by a lab member and coded. The main experimenter conducts the tests and the genotype is revealed afterwards. Most data requiring image analysis (Oil red O, fluorescence intensity), genotype was blinded at the moment of analysis.

Reporting for specific materials, systems and methods

We require information from authors about some types of materials, experimental systems and methods used in many studies. Here, indicate whether each material, system or method listed is relevant to your study. If you are not sure if a list item applies to your research, read the appropriate section before selecting a response.

Materials & experimental systems

n/a	Involved in the study	
☒	☐	Antibodies
☒	☐	Eukaryotic cell lines
☒	☐	Palaeontology and archaeology
☐	☒	Animals and other organisms
☒	☐	Human research participants
☒	☐	Clinical data
☒	☐	Dual use research of concern

Methods

n/a	Involved in the study	
☒	☐	ChIP-seq
☒	☐	Flow cytometry
☒	☐	MRI-based neuroimaging

Animals and other organisms

Policy information about studies involving animals; ARRIVE guidelines recommended for reporting animal research

Laboratory animals	The N2 Bristol strain, as well as EG1285 (oxls12 [unc-47p::GFP + lin-15(+)]), VS24 (kat-1(tm1037)), VC1011 (acdh-1(ok1489)), VC425 (elo-6(gk233)), VL749 (wwls24[Pacdh-1::GFP; unc-119(+)]), RB754 (aak-2(ok524)), CF1038 (daf-16(mu86)), VC199 (sir-2.1(ok434)), PS3551 (hsf-1(sy441)), SJ4197 (zcls39[dve-1p::dve-1::GFP]) , IG274 (frls7[nlp-29p::GFP,col-12p::DsRed]), SJ4100 (zcls13[hsp-6::GFP]), SJ4005 (zcls4[hsp-4::GFP; lin-15(n765)), SJ4058 (zcls9 [hsp-60::GFP + lin-15(+)]), TJ375 (gpls1[hsp-16.2p::GFP]), AM44 (rmls190 [F25B3.3p::Q67::CFP]), AM101 (rmls110 [F25B3.3p::Q40::YFP]) and AM141 (rmls133 [unc-54p::Q40::YFP]) were obtained from the C. elegans Genetics Center (University of Minnesota, Minneapolis), which is funded by NIH Office of Research Infrastructure Programs (P40 OD010440). VC425, VC1011 and VC199 was provided by the C. elegans Reverse Genetics Core Facility at the University of British Colombia, which is part of the international C. elegans Gene Knockout Consortium 145. RB754 was provided by the C. elegans Gene Knockout Project at the Oklahoma Medical Research Foundation, which is also part of the international C. elegans Gene Knockout Consortium 145. FX03278 (acs-20(tm3278)) was obtained from S. Mitani and the Japanese National BioResource Project (Tokyo, Japan). PHX1928 (acdh-10(syb1928)) was made by SunyBiotech Co., Ltd by introducing a nonsense mutation in the gene. Mutant strains were outcrossed to N2 4 times before use. Other C. elegans strains were obtained by crossing. Homozygosity of all genotypes was confirmed by PCR or sequencing.

Transgenic lines expressing mutant TDP-43A315T, wild-type TDP-43 (TDP-43WT), mutant FUSS57 and wild-type FUS (FUSWT) were created as previously described 25. Several strains showing comparable phenotypes and transgene expression levels were kept and the strains used in this study include : XQ98 (xqIs98 [unc47p::FUSS57?;unc-119(+)]), XQ173 (xqIs173 [unc-47p::FUSWT; unc-119(+)]), XQ132 (xqIs132 [unc-47p::TDP-43WT; unc-119(+)]) and XQ133 (xqIs133 [unc-47p::TDP-43A315T; unc-119(+)]).

For samples from G93A mice :
B6SJL-Tg(SOD1*G93A)1Gur/J (JAX, Stock No: 002726), B6SJL-Tg(SOD1)2Gur/J (JAX, Stock No: 002297) and B6SJL mice (for colonie maintenance) were used. Male SOD1G93A progeny were used for further breeding to maintain the line. Only female mice were used for these experiments.

Wild animals	This study did not involved wild animals.
Field-collected samples	This study did not involved field-collected samples.
Ethics oversight	No ethical guidance or approval is required for invertebrate organisms such as nematodes. For samples from G93A mice, animals were treated according to our approved protocols from the CRCHUM Institutional Committee for the Protection of Animals and the Canadian Council on Animal Care (CCAC). All procedures were carried out in accordance with the Guidelines for Preclinical Animal Research in ALS/MND.

Note that full information on the approval of the study protocol must also be provided in the manuscript.
